# Localization of mutant ubiquitin in the brain of a transgenic mouse line with proteasomal inhibition and its validation at specific sites in Alzheimer's disease

**DOI:** 10.3389/fnana.2015.00026

**Published:** 2015-03-17

**Authors:** Romina J. G. Gentier, Bert M. Verheijen, Margherita Zamboni, Maartje M. A. Stroeken, Denise J. H. P. Hermes, Benno Küsters, Harry W. M. Steinbusch, David A. Hopkins, Fred W. Van Leeuwen

**Affiliations:** ^1^Department of Neuroscience, Faculty of Health, Medicine and Life Sciences, Maastricht UniversityMaastricht, Netherlands; ^2^Department of Pathology, Radboud University Nijmegen Medical CenterNijmegen, Netherlands; ^3^Department of Pathology, Maastricht University Medical CenterMaastricht, Netherlands; ^4^Department of Medical Neuroscience, Dalhousie UniversityHalifax, NS, Canada

**Keywords:** frameshift mutation, ubiquitin B^+1^, olfactory bulb, basal ganglia, nucleus basalis of Meynert, inferior colliculus, raphe nuclei, RNA

## Abstract

Loss of protein quality control by the ubiquitin-proteasome system (UPS) during aging is one of the processes putatively contributing to cellular stress and Alzheimer's disease (AD) pathogenesis. Recently, pooled Genome Wide Association Studies (GWAS), pathway analysis and proteomics identified protein ubiquitination as one of the key modulators of AD. Mutations in ubiquitin B mRNA that result in UBB^+1^ dose-dependently cause an impaired UPS, subsequent accumulation of UBB^+1^ and most probably depositions of other aberrant proteins present in plaques and neurofibrillary tangles. We used specific immunohistochemical probes for a comprehensive topographic mapping of the UBB^+1^ distribution in the brains of transgenic mouse line 3413 overexpressing UBB^+1^. We also mapped the expression of UBB^+1^ in brain areas of AD patients selected based upon the distribution of UBB^+1^ in line 3413. Therefore, we focused on the olfactory bulb, basal ganglia, nucleus basalis of Meynert, inferior colliculus and raphe nuclei. UBB^+1^ distribution was compared with established probes for pre-tangles and tangles and Aβ plaques. UBB^+1^ distribution found in line 3413 is partly mirrored in the AD brain. Specifically, nuclei with substantial accumulations of tangle-bearing neurons, such as the nucleus basalis of Meynert and raphe nuclei also present high densities of UBB^+1^ positive tangles. Line 3413 is useful for studying the contribution of proteasomal dysfunction in AD. The findings are consistent with evidence that areas outside the forebrain are also affected in AD. Line 3413 may also be predictive for other conformational diseases, including related tauopathies and polyglutamine diseases, in which UBB^+1^ accumulates in their cellular hallmarks.

## Introduction

Alzheimer's disease (AD) is a multifactorial disease and the most prevalent form of dementia. Currently, it is estimated to affect at least 20 million people worldwide and the prevalence is expected to triple within the next 40 years (Barnes and Yaffe, 2011). AD neuropathology is characterized by two cellular hallmarks: the accumulation of extracellular plaques mainly composed of amyloid β (Aβ) and of intracellular hyperphosphorylated tau in neurofibrillary tangles (NFTs) (Selkoe, 2001). In autosomal dominant AD cases (accounting for <3%) (Van Leeuwen et al., 2000), mutations in three different genes have been identified: the amyloid precursor protein (APP) and the presenilin 1 (PS1) and 2 (PS2) genes. Pathogenic mutations in these genes contribute to aberrations of the γ-secretase complex, which leads to an increased production of toxic Aβ 42 (Jankowsky et al., 2004). In the tau gene of AD patients no mutations have been reported so far. The major risk factor for developing sporadic AD is aging whereas the ε4 polymorphism of the apolipoprotein E gene (APOE) is the most prominent genetic risk factor (Corder et al., 1993). Other medium-risk factors such as a missense mutation in the gene encoding the triggering receptor expressed on myeloid cells 2 (TREM2) (Jonsson et al., 2013) and nine low risk factors from Genome Wide Association Studies (GWAS) were reported to contribute to AD (Holton et al., 2013). Recently, pooled GWAS studies and pathway analysis also identified protein ubiquitination as one of the key modulators of AD (International Genomics of Alzheimer's Disease Consortium (IGAP), 2015). In this study they implicate that the immune response, regulation of endocytosis, cholesterol transport, and protein ubiquitination represent prime targets for AD therapeutics. In addition, a brain site specific strategy was used to compare the proteomes of prefrontal cortex, hippocampus and cerebellum in brains of AD patients (Manavalan et al., 2013). An Ingenuity Pathway Analysis demonstrated 31 proteins were significantly altered and that these proteins had a strong interaction with the ubiquitin C (UBC) signaling pointing to a dysfunctional ubiquitin proteasome system (UPS) as a causative factor in AD (Manavalan et al., 2013).

Neuronal development and synaptic plasticity are part of processes that involve metabolism of 5–8% of brain proteins each day (Dennissen et al., 2012). This turnover requires an efficient protein quality control (PQC) for which the UPS and autophagy are mainly responsible. We discovered that the transcription of the ubiquitin B (UBB) gene can result in accumulation of mutant ubiquitin B^+1^ (UBB^+1^) in the cellular hallmarks (plaques and tangles) of sporadic and autosomal dominant AD cases, suggesting a pathological function (Van Leeuwen et al., 1998, 2006). Similarly, other tauopathies, as well as several polyglutamine diseases (e.g., Huntington's diseases (HD)) are characterized by the accumulation of UBB^+1^ in the respective hallmarks. By contrast, UBB^+1^ appears not to be involved in synucleinopathies (Fischer et al., 2003; De Pril et al., 2004). UBB^+1^ compromises PQC by inhibiting the UPS dose-dependently (Dennissen et al., 2012) via inhibition of deubiquitinating enzymes (DUB) (Krutauz et al., 2014).

*In vivo* studies performed in a transgenic (tg) line (#3413) overexpressing human UBB^+1^, specifically in neurons postnatally, showed increased levels of ubiquitinated proteins in the forebrain (e.g., cerebral cortex, hippocampus, dentate gyrus, amygdala, and striatum). These tg mice show deficits in contextual memory, a decrease in proteasome activity and proteomic changes reminiscent of AD (Fischer et al., 2009). In addition, a comprehensive phenotypic screen of line 3413 revealed a respiratory phenotype (Irmler et al., 2012). Changes in spontaneous breathing patterns and an altered hypoxic response, suggested a central dysfunction of respiratory regulation. In keeping with this, expression of UBB^+1^ was found in the nucleus of the tractus solitarius (Sol) and the parabrachial nuclei, brainstem nuclei involved in respiratory control. These data suggest that respiratory centers in the brainstem are sensitive to long-term UPS inhibition via the expression of the UBB^+1^ protein. Most interestingly, UBB^+1^ immunoreactivity in AD patients was seen in similar areas as in the tg mice, suggesting a possible functional link between UBB^+1^ expression in brainstem areas and the respiratory and swallowing dysfunctions that are often seen in AD patients (Irmler et al., 2012). Moreover, in early stages of AD, it has been noted that increased cardiorespiratory (CR) fitness in early-stage AD is associated with reduced brain atrophy as compared with non-demented individuals (Burns et al., 2008). It was also shown that declining CR fitness over 2 years was associated with brain atrophy, especially in the parahippocampus in AD (Vidoni et al., 2012). Dysphagia is also an issue in Parkinson's disease (PD) caused by synucleinopathology in the glossopharyngeal nucleus (Braak et al., 2003; Cereda et al., 2014), starts initially in the dorsal motor nucleus of the vagus nerve (Braak et al., 2003) but it is UBB^+1^ negative.

Previous studies have concentrated mainly on parts of the forebrain and the brainstem; however, information about UBB^+1^ accumulation in other brain areas of these tg mice and the possible functional consequences of UBB^+1^ expression has been lacking. The pattern of UBB^+1^ expression and expression levels in the tg mice are largely dictated by copy numbers and CamKIIα promotor elements used to drive expression, therefore the results of the tg mice must not be overestimated (Fischer et al., 2009). The aim of the present study is to provide a comprehensive topographic mapping of UBB^+1^ in the brains of the tg mouse line 3413. Due to the extensive UBB^+1^ expression in certain mouse brain areas, a next step was to compare this distribution with the distribution of UBB^+1^ and established markers of AD in the human brain. This approach identified two immunoreactive brainstem areas that show a similar immunoreactivity in AD and could be linked to respiratory dysfunction (Irmler et al., 2012). As we detected additional high intensities of UBB^+1^ immunoreactivity, especially in five other brain areas in line 3413, we focus here on these brain areas known to be affected in AD, namely the olfactory bulb (OB), basal ganglia, nucleus basalis of Meynert (NBM), raphe nuclei and inferior colliculus (IC) and discuss their potential relevance for AD research.

## Materials and methods

### Animals

In the present study, UBB^+1^ tg male mice (line 3413,008833C57Bl/6.Tg(CaMKIIα-UBB) 3413, Jackson #008833) (*n* = 14; eight 3-month-old mice, two 7-month-old mice, and four 15-month-old mice) expressing human UBB^+1^ in the postnatal brain on a pure C57BL/6 background were used (Fischer et al., 2009). UBB^+1^ cDNA is encoded by the first ubiquitin upstream open reading frame and a murine calmodulin kinase II alpha (CaMKIIα) promoter was used to regulate the UBB^+1^ expression in the tg mice (Van Leeuwen et al., 1998). Non-transgenic littermates were used as controls (*n* = 9). To ensure comparability among individuals, mice were kept under standard animal housing conditions: a 12/12 h light- dark cycle with food and acidified water *ad libitum* in specific pathogen free conditions.

All animals used in the present study were males and were sacrificed at either 3, 7 and 15 months of age. All animal experiments were performed conforming to national animal welfare law and under guidance of the animal welfare committees of the Royal Netherlands Academy of Arts and Sciences and of Maastricht University (Dier Experimenten Commissie (DEC/Animal Experiments Commission) protocol nr. 2008-069).

### Immunohistochemistry

Mice were deeply anesthetized with sodium pentobarbital and were perfused transcardially with Tyrode solution (2.68 mM KCl, 0.245 mM MgCl_2_.6H_2_O, 136 mM NaCl, 0.289 mM NaH_2_PO_4_.H_2_O, 5 mM glucose, 12 mM NaHCO_3;_pH 7.4) aerated with carbogen followed by 0,1M phosphate buffer containing 4% paraformaldehyde (pH 7.4). Brains were removed and placed in fixative overnight on a rocking table at 4°C. Subsequently, they were stored in a 1% sodium azide (NaN_3_) phosphate-buffered saline (PBS) solution in a cold room at 4°C until further processing. Brains were embedded in gelatin and sectioned on a Vibratome (Leica VT 1200S, Wetzlar, Germany) into 50 μm coronal or sagittal free-floating sections. Tissue sections were stained overnight at 4°C (first 1 h at room temperature (RT)) with a primary polyclonal rabbit anti-UBB^+1^ antibody (Ubi3 16/09/97, final dilution 1:1000), recognizing the C-terminal extension of the UBB^+1^ protein (Fischer et al., 2003). The sections were incubated with a biotinylated donkey anti-rabbit antibody (1:400) (Jackson Laboratories) followed by avidin-biotin-peroxidase (ABC,1:400, Vector), both 1 h at RT. The staining was visualized with 3,3′-diaminobenzidine tetrahydrochloride (DAB) solution intensified by 0.2 % nickel ammonium sulfate (pH7.6). The sections were mounted on gelatin-coated glass slides, air dried, dehydrated, and coverslipped using Pertex (Histolab). As controls for Ubi3 specificity, Ubi3 antiserum adsorbed with ^6^His tag UBB^+1^ as well as the pre-immune control serum were used.

Human postmortem tissue (OB, basal ganglia, NBM, IC, and raphe nuclei) from AD patients and non-demented controls was obtained from the Radboud University Medical Center (Department of Pathology, Nijmegen, The Netherlands, Table 1). Anonymised human material from AD-patients and non-demented control patients was used according Dutch law and local guidelines. The number of available human tissue material of the patients is different for each of the focused brain areas. The tissues were fixed in 4% buffered paraformaldehyde for at least 3 weeks. Part of the human OB tissue specimens were cryoprotected in 10% sucrose solution (0.1 M phosphate buffer, pH 7.6, at 4°C) followed by at least 72 h incubation in 20% sucrose solution. Subsequently, this tissue was frozen and stored at −80°C. Coronal sections of 8μm thickness were mounted on gelatin-coated glass slides and stored at −80°C until further processing. The other OB tissue specimens with olfactory cortex added were dehydrated, embedded in paraffin and cut in the sagittal plane, producing serial sections of 8 μm thickness each. Tissue of the basal ganglia, NBM, IC, and raphe nuclei were fixed in 4% buffered paraformaldehyde for 1 month after which the unembedded tissue was cut on a Vibratome in 50 μm thick sections and stored at 4°C in PBS with 1% NaN_3_until further processing. Basal ganglia were cut in the coronal plane, whereas brainstem (IC and raphe nuclei) was cut perpendicular to the long axis of the spinal cord, to take into account the flexures of the human neuraxis.

**Table 1:** **Clinico-pathological information of non-demented controls and AD patients**.

Case^*^	**Age (years)**	**Sex (f/m)**	**Braak stage^a^**	**Amyloid^a^**	**Brain Weight (g)**	**Cause of dealth**	**Olfactory bulb**	**Basal ganglia^v^**	**Inferior Colliculus^v^**	**Raphe Nuclei^v^**	Brainstem^**^	
											**NTS^v^**	**PBN^v^**
1	52	m	0	0	1424	Cardiac infarction	+^c^	−	−	−	+	+
2	59	f	0	0	1523	Cardiac infarction lung oedema	−	+	+	+	+	+
3	73	m	0	0	1474	Unknown	+^p^	+	+	+	−	−
4	87	m	3	Sparse	1245	Cardiorespiratory failure with cardiac decompensation, signs of pneumonia and sepsis	+^c^	+	+	+	+	+
5	64	m	5	Frequent	1123	Cardiorespiratory insufficiency	+^p^	+	+	+	−	−
6	66	m	5	Frequent	1360	Unknown	+^p^	+	+	+	−	−
7	68	m	5	Frequent	1312	Subcortical bleeding	−	+	+	+	+	+
8	90	f	5	Frequent	1118	Asystolic	+^p^	+	+	+	−	−
9	64	f	6	Frequent	1250	Cachexia, dehydration	+^c^	−	−	−	+	+
10	84	f	6	Frequent	1090	Uraemia, dehydration, respiratory tract infection	+^c^	−	−	−	+	+

a *, based upon examination of temporal cortex and hippocampus*.

p *, paraffin-embedded sections of the olfactory bulb and olfactory cortex (8 μm)*.

c *, cryostat sectioned olfactory bulb and olfactory cortex tissue (10 μm)*.

v *, Vibratome sectioned brain tissue (50 μm)*.

* *All patients had a postmortem delay between 24 and 48 h and a fixation time of at least 3 months*.

** *Brainstem data reported in Irmler et al. (2012)*.

*−, no tissue available*.

To perform immunohistochemistry, the frozen sections and the paraffin-embedded sections (after deparaffination) were incubated for 30 min in 100% formic acid followed by rinsing in distilled water (30 min) and in Tris-buffered saline (TBS) (30 min) and subsequently incubated overnight at 4°C (first 1 h at RT) with antibodies against misfolded tau (monoclonal MC1, 1:100, Dr. P. Davies, New York) (Petry et al., 2014), phosphorylated tau serine 202 (monoclonal CP13, 1:100 Dr. P. Davies, New York)(Petry et al., 2014), amyloid β (monoclonal 6F3D,1:100, Dako) and UBB^+1^ (polyclonal Ubi2A, 180398, 1:400 or polyclonal Ubi2^+1^ 140994, 1;400). All dilutions were in Sumi buffer (0.05 M Tris with 0.9% NaCl, 0.25 M gelatin and 0.5% Triton X-100, pH 7.4) (Fischer et al., 2003; Van Leeuwen et al., 2006). Next, sections were incubated with secondary donkey anti-mouse or donkey anti-rabbit antibodies, both biotinylated (1:400, Jackson Laboratories) and ABC (1:400, Vector), both 1 h at RT and finally stained with DAB, dehydrated and coverslipped as described above.

Free-floating Vibratome sections were pretreated with 100% formic acid for the 6F3D antibody for 30 min and then rinsed in distilled water (30 min) and TBS (10 min). Subsequently, all sections were treated with graded series of methanol (20, 40, 60, 80% (10 min) and 100% (30 min), and back to 20% methanol with 0.3% H_2_O_2_. Rinsing was performed for 30 min in TBS followed by 30 min in Sumi buffer. Sections were then incubated with a primary antibody (1 h at RT followed by 36 h incubation at 4°C in a humid chamber), biotinylated secondary antibodies (2 h at RT), ABC (2 h at RT), and DAB as described above. All sections were mounted on gelatin-coated glass slides, air dried, dehydrated and coverslipped with DPX (Klinipath). As controls for Ubi2^+1^ and Ubi2A specificity, Ubi2^+1^ and Ubi2A antiserum adsorbed with ^6^His tag UBB^+1^ as well as the pre-immune control serum were used (Van Leeuwen et al., 1998; Fischer et al., 2003).

### Microscopy

Brain sections of 3413 tg and control mice were analyzed semi-quantitatively using an atlas (Franklin and Paxinos, 2007) and light microscopy, by focusing on intensity (I) of individual neuron staining and density (D) of relative numbers of stained neurons showing UBB^+1^ immunoreactivity (ir) in a specific brain nucleus. Intensity is defined as the stain quality in a specific brain region while density is defined as the number of UBB^+1^ immunoreactive cells in this region. Scoring was assessed independently by two observers: - no UBB^+1^ ir, + a low I or D of UBB^+1^-ir cells, ++ a moderate I or D of UBB^+1^-ir cells, +++ a high I or D of UBB^+1^-ir cells, and ++++ a very high I or D of UBB^+1^-ir cells. The mean I and D per brain nuclei were determined semi-quantitatively for final results. Based on these scores, five areas with a high expression of UBB^+1^ were selected for the analysis of the human tissue.

For the human sections a similar semi-quantitative approach was applied for the light-microscopic analysis. Layers of the OB were defined following indications provided in (Mai and Paxinos, 2012).

Subdivisions of basal ganglia (Mai et al., 2008) and brainstem nuclei (Haines, 2011) were identified, using atlases, based upon their topographical position. Figure S1 illustrates micrographs of sections used for the analysis, taken at the levels of basal ganglia and mesencephalon showing corresponding subdivisions in nuclei and white matter regions. The presence of AD-related neuropathology was estimated semi-quantitatively as − (negative), + (low), ++ (moderate), and +++ (high) based on densities of NFTs, plaques and positive neuronal cells. In addition, qualitative descriptions were made on the distribution of neuropathology and the morphological features within the structures. Specifically, a classification of immunoreactive substrates was made upon the categorization proposed by Duyckaerts et al. (2009). Amyloid deposits were identified as neuritic plaques when they presented a tau-positive corona of dystrophic neurites or as plaques when such neuritic components were absent. Tau-immunoreactivity was further identified as neuropil threads (NTs) when stained substrates had the form of small, tortuous processes and as NFTs when immunoreactivity also present in neuronal somata. Photographs were made using a dotSlide BX51microscope (Olympus, Japan).

## Results

The main goals of the present study were to describe the distribution of UBB^+1^ in the brain of a UBB^+1^ tg mice model compared to control mice and to compare the distribution to that in homologous human brain areas. One can observe high or intense staining of individual neurons and a low density of intensely stained neurons and vice versa. No differences in immunoreactivity were found among 3-, 7-, and 15-month-old 3413 tg mice (Table 2, Table S2) and UBB^+1^ was absent in all brain regions of the control mice (Van Tijn et al., 2011). The incubation of tg mouse brain tissue with ^6^His tag UBB^+1^ and the pre-immune control serum resulted in an absence of immunopositive staining (Irmler et al., 2012). Relative UBB^+1^ immunoreactivity scores for intensity and density in the mouse brain are shown in **Figure 2**. Abbreviations of anatomical brain structures are listed in alphabetical order in the list of abbrevations and in Table S1. In the next paragraphs we will discuss first the results of the mouse line 3413 brain anatomy followed by the results of the human immunohistochemical study.

**Table 2:** **UBB^+1^ immunoreactivity in the cerebral cortex, hippocampus, dentate gyrus, striatum, amygdala, nucleus parabrachialis, locus coeruleus, nucleus of the tractus solitarius, olfactory area, basal ganglia, nucleus basalis of Meynert, auditory area and in the raphe nuclei of 3413 tg mice**.

**Brain nuclei**	**3413 UBB^+1^I**	**3413 UBB^+1^ D**
**CEREBRAL CORTEX**		
Apir	++	++
Cent	+++	+++
Cg1	++	++
Cg2	++	++
Cortical L1	−	−
Cortical L2	++	+++
Cortical L3	+	+++
Cortical L4	+	+++
Cortical L5a	+	+
Cortical L5b	++	++
Cortical L6	+	++
CxA	+	++
Den	++	++
DP	+	++
Ect	++	+++
FrA	++	++
IL	+	+
LO	++	++
M2	++	++
Pir	++	++
PrL	+	++
RSD	++	++
RSGa	+++	++
RSGb	+++	++
RSGc	+++	++
V1	+++	+++
V2MM	++	++
V2ML	−	−
Ven	++	++
VO	++	++
**HIPPOCAMPUS**		
CA1	+++	++++
CA2	+++	++++
CA3	+++	+++
FC	++	+++
Ig	++	+++
Lmol	−	−
Mol	−	−
PaS	++	++
PrS	++	++
Py	+++	++++
Rad	−	−
Sb	++	++
Shi	++	+++
Slu	+++	++++
**DENTATE GYRUS**		
PoDG	++	++
GrDG	+++	++++
**AMYGDALA NUCLEI**		
AAD	+	+
AAV	+	+
Aco	−	−
Ahi	+	++
AhiAL	+	++
AhiPM	++	++
Astr	++	++
BLA	+	++
BLP	++	++
BLV	+	++
BMA	+	++
BMP	+	++
CeC	++	++
CeL	++	++
CeM	+	++
CeMAD	+	++
CeMAV	+	++
CeMPV	+	++
I	++	+
IM	+	++
La	++	++
LaDL	++	++
LaVL	++	++
LaVM	+	+
MeA	+	++
MeAD	++	++
MeAV	++	++
MePD	++	++
MePV	+	++
PLCo	++	++
PMCo	+	++
SLEA	+	+
SLEAC	+	+
SLEAM	+	++
**NUCLEUS PARABRACHIALIS**		
LPB	+++	+++
LPBC	+++	++
LPBD	++++	+++
LPBE	++++	+++
LPBI	++	++
LPBV	++	++
MPB	+++	++
MPBE	++	++
PBW	+	++
**LOCUS COERULEUS**		
LC	+	++
**NUCLEUS TRACTUS SOLITARIUS/AREA POSTREMA/DORSAL MOTOR NUCLEUS OF THE VAGUS NERVE**		
Psol	+	++
Sol	++	++
SolC	++	++
SolCe	+	++
SolDL	++	++
SolDM	+	++
SolG	++	++
SolI	+	++
SolIM	++	++
SolM	+	++
SolV	+	++
SolVL	+	++
AP	+++	++
10N	++	++
**OLFACTORY AREAS**		
AOB	++	++
AOD	+	++
AOE	++	++
AOL	+	++
AOM	+	++
AOP	+	++
AOV	+	++
BAOT	++	++
DTT	+	++
Epl	++	+
E/OV	+	++
Gl	++	+
GIA	++	+
GrA	+	++
GrO	+	+
Ipl	+	+
LOT	++	++
Mi	+	+
MiA	++	++
VTT	++	++
**BASAL GANGLIA**		
**Striatum**		
Cpu	++	++
LSS	+	++
**Nucleus accumbens**		
Acb	+	+++
AcbC	++	+++
AcbSh	++	+++
LacbSh	++	+++
**Islands of Calleja**		
Icj	++	++
IcjM	+	++
**Olfactory tubercle**		
Tu	++	++
**Globus Pallidus**		
LGP	−	−
MGP	−	−
**Subthalamic nucleus**		
STh	+	+
**NUCLEUS BASALIS OF MEYNERT**		
NBM	+	++
**AUDITORY SYSTEM NUCLEI**		
**Inferior colliculus**		
BIC	+	++
CIC	+	++
DCIC	++	+++
ECIC	+	+++
ECIC L1	++	+++
ECIC L2	++	+++
ECIC L3	+	+++
**Medial Geniculate Nucleus**		
MGD	+	++
MGV	+	+
MGM	+	+
MZMG	+	+
**Cochlear Nuclei**		
DC	+++	+
GrC	−	−
VCA	+	+
VCP	−	−
**Trapezoid nucleus**		
Tz	−	−
**Superior olive**		
LSO	+	+
**RAPHE NUCLEI**		
Cli	+	+
DR	+++	++
DRC	+++	++
DRD	+++	+++
DRI	+++	+++
DRV	++	++
DRVL	++	++
MnR	++	++
PMnR	++	++
PnR	++	++
RC	+	++
Rli	−	−
RMg	+++	++
Rob	+	+
Rpa	+	+

*Relative UBB^+1^ immunoreactivity scores for intensity and density are illustrated in Figure 2. No UBB^+1^ immunoreactivity was present in any brain regions in the WT mice*.

*−, No immunoreactivity; +, low I or D; ++, moderate I or D; +++, high I or D; ++++, very high I or D*.

### Mouse studies

#### UBB^+1^ in the telencephalon and diencephalon

**Figure 1:**
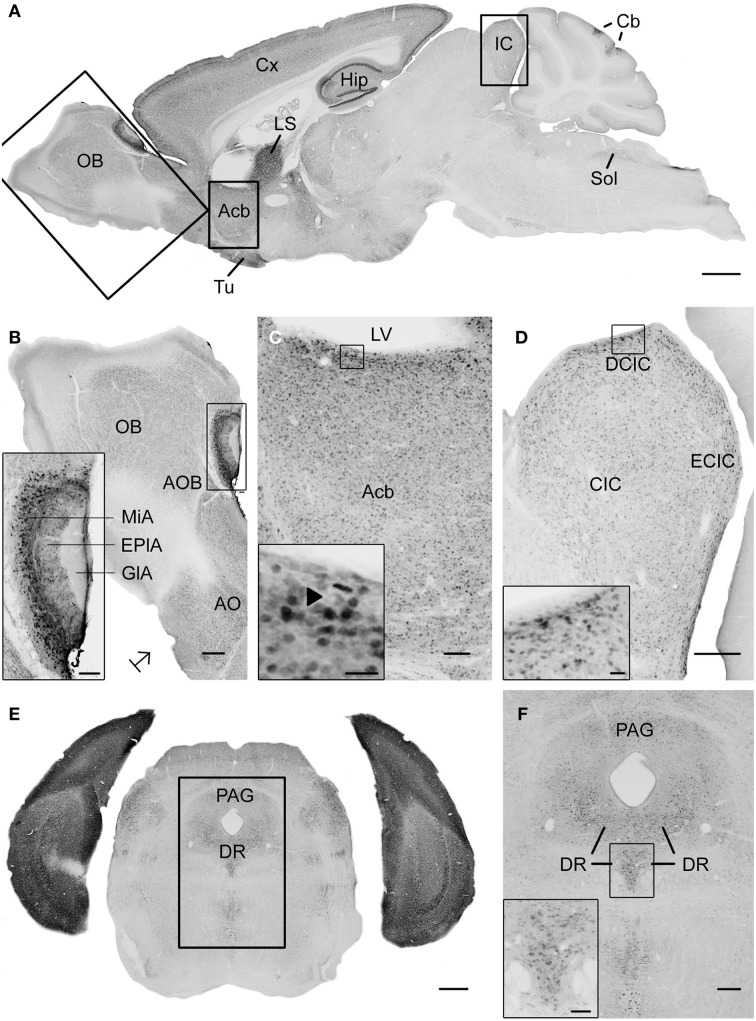
**Photomicrographs of UBB^+1^ distribution in the brain of UBB^+1^ tg mice (line 3413) shown in sagittal and coronal sections. (A)** Sagittal overview of UBB^+1^ staining in a 7-month-old 3413 tg mouse corresponding to sagittal figure 106 in the mouse brain atlas of Franklin and Paxinos (2007) with rectangles showing the locations of higher magnifications in **(B–D)**. **(B)** OB, **(C)** Acb and **(D)** IC. **(B)** Shows the presence of UBB^+1^ in neurons of the OB, accessory olfactory bulb (AOB) and the anterior olfactory area (AO) at higher magnification. The insert shows a higher magnification of the AOB. **(C)** UBB^+1^ expression in the Acb. Insert shows higher magnification of UBB^+1^-positive cells. *Filled triangle* shows a cell in which the UBB^+1^ staining is cytoplasmic and the nucleus is negative. **(D)** UBB^+1^-immunoreactivity in the IC. Insert shows a higher magnification of the immunoreactive cells in the DCIC. **(E)** Coronal section of a 15-month-old 3413 tg mouse showing the presence of UBB^+1^ in the dorsal raphe (corresponding to coronal figure 69 in the mouse brain atlas of Franklin and Paxinos, 2007). **(F)** shows this region at a higher magnification. Insert in **(F)** shows the UBB^+1^-immunoreactive cells in the DR. Bars: **(A)**, 1 mm; **(B,D,F)**, 200 μm, (insert in **B,C**), 100 μm, **(E)**, 500 μm, (insert in **C**, insert in **D**), 20 μm, insert in **(F)**, 50 μm. Acb, nucleus accumbens; AOB, accessory olfactory bulb; AO, anterior olfactory area; Cb, cerebellum; CIC, central nucleus of IC; Cx, cerebral cortex; DCIC, dorsal cortex of IC; DR, dorsal raphe; EplA, external plexiform layer of the accessory olfactory bulb, ECIC, external cortex of IC; GlA, glomerular layer of the accessory olfactory bulb; Hip, hippocampus; IC, inferior colliculus; LS, lateral septal nucleus; LV, lateral ventricle; MiA, mitral cell layer of the accessory olfactory bulb; OB, olfactory bulb; PAG, periaqueductal gray; Sol, nucleus of the tractus solitarius; Tu, olfactory tubercle.

**Figure 2:**
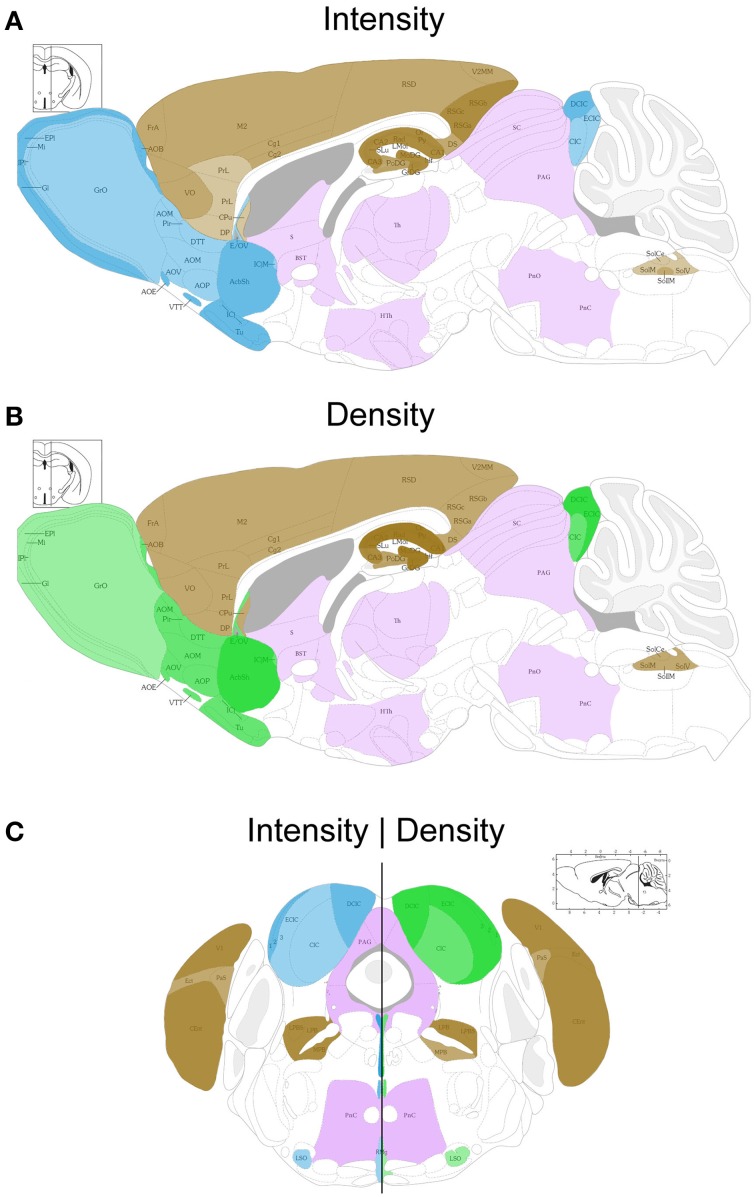
**Schematic sagittal (A,B) and coronal (C) overviews showing the mean UBB^+1^ intensity (blue) (A,C left) and density (green) (B,C right) in a wide range of mouse brain nuclei**. Regions previously reported to be UBB^+1^ immunoreactive (cerebral cortex, hippocampus, dentate gyrus, amygdala, striatum, nucleus parabrachialis, and Sol) are shown in brown. The UBB^+1^ intensity and density in OB, Acb, IC and the raphe nuclei are shown in blue and green, respectively. Different gradations of brown, blue, and green are used indicating the level of staining intensity or density with a light gradation for a low intensity or density, a medium gradation for a moderate intensity or density level, a dark gradation for a high intensity or density level and a very dark gradation for a very high intensity or density. Other sizeable regions with a low to moderate UBB^+1^-immunoreactivity are shown in purple. However, more regions are positive for UBB^+1^, for details see Tables 2 and S2. Figures are adapted from the mouse brain atlas of Franklin and Paxinos figure 106 and 73, respectively (Franklin and Paxinos, 2007). BST, bed nucleus of the stria terminalis; Gi, gigantocellular reticular nucleus; HTh, hypothalamus; PAG, periaqueductal gray; PnC, pontine reticular nucleus, caudal part; PnO, pontine reticular nucleus, oral part; S, septal area; SC, superior colliculus; Th, thalamus.

The present study extends and provides specific details in follow up to our initial global descriptions of UBB^+1^ expression in the forebrain (e.g., cerebral cortex, hippocampus, dentate gyrus, amygdala, and striatum) and the brainstem in the 3413 tg mice (Fischer et al., 2009; Irmler et al., 2012) (Figures 1, 2, Table 2). The CaMKIIα promoter mainly results in strong protein expression in forebrain areas (Mayford et al., 1996a). In keeping with this, the expression of UBB^+1^ protein was strong in the forebrain. In addition, after extensive mapping we observed a wide range of UBB^+1^ expression in hindbrain regions (e.g., brainstem and cerebellum) where other groups also showed the presence of CamKIIα (Mayford et al., 1996b). The analysis of the immunohistochemical expression in the mouse brain is aggregated by anatomical region (i.e., telencephalon, diencephalon, mesencephalon, pons, medulla oblongata, and cerebellum).

#### Telencephalic structures in the mouse brain

In most of the forebrain regions, the 3413 mice showed low to very high expression (+; ++; +++; ++++) of UBB^+1^ (Table 2, Figure 2). In the neocortex, layers (L) 2, 3, 4, 5, and 6 showed a low to moderate intensity with a moderate (L5, L6) to high (L2, L3, L4) density of UBB^+1^ cells while UBB^+1^ was absent in L1. These data demonstrated that UBB^+1^ was not uniformly stained in the classical six layers of the neocortex. The caudomedial entorhinal cortex (CEnt), retrosplenial granular cortex (RSGa, b and c) and primary visual cortex (V1) showed a high UBB^+^-intensity while the other cortical areas (e.g., APir, Cg1, Cg2, Pir, CxA, DEn, DP, Ect, FrA, IL, LO, M2, PrL, RSD, V2MM, VEn, and VO) varied between a low to moderate intensity (Table 2). A moderate density was present in all the cortical areas except for infralimbic cortex (IL) with a low density and the CEnt, ectorhinal cortex (Ect) and V1 which showed a high density of UBB^+1^ cells. The mediolateral area of the secondary visual cortex (V2ML) was UBB^+1^-negative (Table 2).

UBB^+1^ neuronal immunoreactivity was distributed throughout the hippocampal region with a variable intensity ranging from moderate to high and a high to very high cell-density. However, the molecular layer of the dentate gyrus (Mol), the stratum lacunosum-moleculare (Lmol) and the stratum radiatum (Rad) were UBB^+1^-negative. The polymorph layer (PoDG) of the dentate gyrus showed a moderate UBB^+1^ intensity and density while the granular layer (GrDG) was highly UBB^+1^ positive with a very high density. (Table 2, Figure 2).

In several amygdala nuclei, a low to moderate intensity and a moderate density of UBB^+1^-ir cells was observed. In the anterior cortical amygdaloid nucleus (ACo) little or no UBB^+1^-immunoreactivity was observed (Table 2). Other telencephalic structures are described in the Supplementary Materials.

The neuronal cells in OB and the accessory olfactory bulb (AOB), both receiving olfactory primary afferents, contain a noticeable amount of UBB^+1^ within a moderate number of positive neurons (Table 2, Figures 1, 2). The OB is subdivided in certain regions which all showed UBB^+1^-immunoreactivity. Specifically, the glomerular layer (Gl) and external plexiform layer (EPl) of the OB showed a moderate UBB^+1^-intensity and a low density. A low intensity and density was present in the mitral cell layer (Mi), in the internal plexiform layer (IPl) and in the granule cell layer (GrO) of the OB. The ependymal and subendymal layer/olfactory ventricle (E/OV) had low intensity UBB^+1^-immunoreactivity with a moderate density of positive neurons. A moderate intensity and density were observed in the nucleus of the lateral olfactory tract (LOT). In the AOB, the mitral (MiA) and glomerular (GIA) cell layer of the AOB expressed a moderate UBB^+1^-intensity while a low intensity was detected in the granule cell layer (GrA) of the AOB. All subdivisions of the AOB showed a moderate density. The anterior olfactory area is subdivided into several anterior olfactory nuclei: dorsal (AOD), external (AOE), lateral (AOL), medial (AOM), posterior (AOP), ventral (AOV). The AOE expressed a moderate UBB^+1^-intensity while the AOD, AOL, AOM, AOP and AOV showed a low intensity. A moderate density was present for all the different nuclei of the anterior olfactory area.

The basal ganglia structures in the telencephalon as reported previously were also immunoreactive for UBB^+1^. The neostriatum [caudate nucleus/putamen (CPu)] showed a moderate intensity and density of UBB^+1^-ir cells. A specific subpopulation of large neurons in CPu is very highly stained for UBB^+1^ while smaller neurons are rather moderate UBB^+1^ immunoreactive (Table 2). In addition to the neostriatum, the nucleus accumbens (Acb), the olfactory tubercle (Tu), and the Islands of Calleja (ICj) showed a moderate to high density of UBB^+1^ positive cells (Table 2, Figures 1, 2). The globus pallidus lateralis (LGP) and medialis (MGP) were UBB^+1^-negative (Table 2). Another basal ganglia component, the subthalamic nucleus (STh) located in diencephalon, showed a low expression for UBB^+1^. Adjacent to the basal ganglia, the UBB^+1^ expression was low in the NBM in tg line 3413 with a moderate number of positive cells (Table 2).

#### Diencephalic structures in the mouse brain

A detailed summary of the UBB^+1^ expression in the diencephalic structures is given in the Supplementary Materials.

#### UBB^+1^ in the mesencephalon

#### Mesencephalic structures in the mouse brain

We analyzed the UBB^+1^ expression in tectal and tegmental mesencephalic brain regions. The IC contained a clear UBB^+1^-immunoreacitiviy. The IC is subdivided into the dorsal cortex (DCIC) and external cortex (ECIC), the central nucleus (CIC) and the nucleus of the brachium of the IC (BIC) which showed staining intensities varying from low to moderate and densities from moderate to high number of UBB^+1^-ir cells (Table 2, Figures 1, 2). The IC is connected with brain structures located in the diencephalon, pons and medulla oblongata, and which are all part of the auditory system e.g., medial geniculate nucleus (MG; diecenphalon), the medullary cochlear nuclei (DC, VC; medulla oblongata), the pontine superior olive and the pontine trapezoid nucleus (Tz; pons). The subnuclei of the medial geniculate nucleus (dorsal, ventral, medial part and the marginal zone) expressed a low UBB^+1^ intensity and density (Table 2). The DC showed a high cell-intensity while the staining in the VC and superior olive was rather low. No immunoreactiviy was detected in the Tz (Table 2).

Several raphe subnuclei are also located in the mesencephalon: caudal (Cli) and rostral (Rli) linear nuclei of the raphe, raphe cap (RC), median (MnR) and paramedian (PMnR) raphe and all the subnuclei of the dorsal raphe nucleus (DR) namely: caudal part (DRC), dorsal part (DRD), interfascicular part (DRI), ventral part (DRV), ventrolateral part (DRVL). The RC and Cli expressed a low UBB^+1^ staining while the Rli was negative for UBB^+1^. A moderate intensity and density was present in the MnR and PMnR. The different subnuclei of the dorsal raphe showed intensities and densities varying from moderate to high (Table 2, Figures 1, 2).

Additional mesencephalic structures and their immunoreactivity for UBB^+1^ are summarized in the Supplementary Materials.

#### UBB^+1^ in the pons, cerebellum and medulla oblongata

#### Pons and cerebellum structures in the mouse brain

As outlined in the introduction, previous experiments examined the presence of UBB^+1^ in respiratory nuclei (Irmler et al., 2012). Table 2 and Figure 2 show a high to very high UBB^+1^-intensity in the nucleus parabrachialis, a pontine respiratory control center. Differences were present in medial external (MPBe), lateral ventral (LPBV), and in lateral internal (LPBI) parabrachial nucleus which have a moderate intensity, and the waist part (PBW), with a low intensity of UBB^+1^-ir cells. The UBB^+1^ density in the different parabrachial nuclei varied from moderate to high. The locus coeruleus (LC) exhibited a similar low to moderate staining intensity (Irmler et al., 2012). The ventral part of the nucleus subcoeruleus showed a low UBB^+1^ expression as well while the dorsal part was negative (Table S2). The staining in the pontine part of the raphe nuclei (PnR) was moderate for intensity and density (Table 2, Figure 1). A more detailed overview about pontine nuclei and the UBB^+1^-immunoreactivity is given in the Supplementary Materials.

The cerebellum showed a high expression of UBB^+1^ in specific cerebellar regions and will be addressed in a separate study.

#### Medulla oblongata in the mouse brain

As reported previously, high UBB^+1^ expression occurred in brain stem centers namely the nucleus of the solitary tract (Sol), the area postrema (AP) and the dorsal motor nucleus of the vagus nerve (10N) (Irmler et al., 2012) (Table 2, Figure 2).

With respect to the medullary raphe nuclei, the raphe obscurus (ROb), and the raphe pallidus (RPa) showed a low UBB^+1^ staining while the raphe magnus (RMg) showed a high intensity and a moderate density (Table 2). More medullary structures positive for UBB^+1^ are described in the Supplementary Materials.

### Human studies

#### UBB^+1^ in the human olfactory area, basal ganglia, and nucleus basalis of meynert

**Figure 3:**
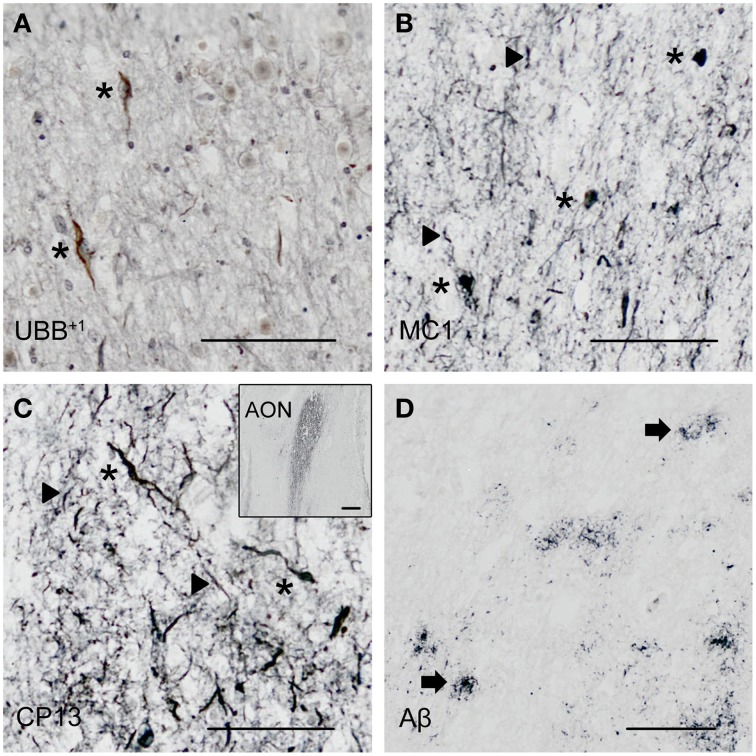
**Expression of UBB^+1^ and AD neuropathology (NFTs, NTs and Aβ) in sections of the human olfactory bulb and tract of patient #10 (Braak 6). (A–D)** shows olfactory tissue incubated with antibodies against **(A)** UBB^+1^, **(B)** pre-tangles (MC1), **(C)** tangles (CP13), and **(D)** Aβ plaques. *Asterisk* shows intracellular accumulations, represented by neuronal staining **(A)** UBB^+1^ immunoreactivity present in neuronal cells of the OB (*asterisk*). **(B–C)** Presence of misfolded tau in pretangles and NFTs *(asterisk)*. NTs are shown by the *filled triangles*. **(C)** The insert shows the AON of the tract. **(D)** Presence of Aβ plaque formation *(arrow)*. Bars: **(A–D)**, 100 μm, insert in **(C)**, 500 μm. AON, anterior olfactory nucleus.

**Figure 4:**
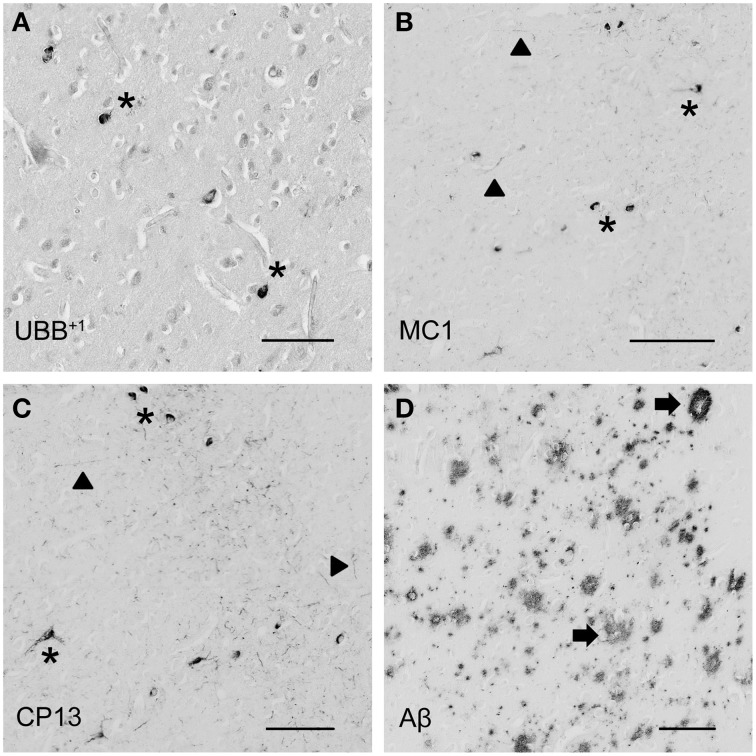
**Photomicrographs of UBB^+1^ and AD neuropathology (NFTs, NTs, and Aβ plaques) in the human piriform (Pir) cortex adjacent to the OB of patient #5 (Braak 5). (A–D)** shows the olfactory tissue incubated with antibodies against **(A)** UBB^+1^, **(B)** pre-tangles (MC1), **(C)** tangles (CP13), and **(D)** Aβ plaques. *Asterisk* shows intracellular accumulations, represented by neuronal staining. **(A)** The presence of UBB^+1^ in the neuronal cells *(asterisk)* of the Pir. **(B,C)** Pir expresses misfolded tau in pretangles and NFTs *(asterisk)*. NTs are shown by *filled triangles*. **(D)** Abundant present of Aβ plaque formation *(arrow)* in the Pir. Bars: **(A–D)**, 100 μm.

The olfactory areas analyzed comprised olfactory bulb, olfactory tract and the cerebral cortical area adjacent to the bulb and tract, piriform cortex (Pir). In order to investigate whether the distribution of UBB^+1^-immunoreactivity overlaps with other pathological changes typical of AD, these areas were also mapped using antibodies against neuronal markers for pre-tangles, NFTs and plaques. These immunohistochemical results showed that AD markers (NFTs and plaques) were present in all AD cases, but not in the non-demented controls (Table 3, Figures 3, 4). A clear differential distribution of pathology was detected within the olfactory system of the Braak 5 cases. The Pir showed high density of immunoreactive substrates (Figure 4), which progressively decreased in the olfactory tract and the bulb. Specifically, the olfactory tract expressed moderate AD-related pathology, and immunoreactivity was restricted to island in the central portion of the structure. This region has been identified by Del Tredici et al. as the anterior olfactory nucleus (AON) (Figure 3) (Del Tredici et al., 2002). In the OB, either no pathology or very few reactive substrates were present. The Pir exhibited both Aβ deposition and tau pathology (Figure 4). High densities of plaques, both neuritic and non-neuritic, were homogeneously distributed throughout the layers of the Pir. NFTs and NTs were present in moderate to high densities and showed a fairly consistent laminar distribution, being concentrated in middle and deep layers.

Table 3**Immunoreactivity for UBB^+1^, pre-tangle material (MC1), tangles (CP13) and plaques (6F3D) in paraffin and cryostat sections of the human olfactory system of controls (Braak stage 0), intermediate phase (Braak stage 3) and AD patients (Braak stage 5)**.

**Table:** 

**Case**	**Olfactory bulb**				**Olfactory tract**				**Piriform cortex**			
	**UBB^+1^**	**MC1**	**CP13**	**6F3D**	**UBB^+1^**	**MC1**	**CP13**	**6F3D**	**UBB^+1^**	**MC1**	**CP13**	**6F3D**
**BRAAK 0**												
3	NA	NA	NA	−	NA	NA	NA	−	−	−	−	−
**BRAAK 5**												
5	−	−	+^*^	−	−	+^*^	++^*^	−	+	++	+++	+++
6	−	−	−	+	−	+^*^	+^*^	−	−	++	++	+++
8	NA	NA	NA	NA	−	+^*^	++^*^	+	+	++	+++	+++

**Table:** 

**Cryoprotected sections of the olfactory bulb**																
**Case**	**Olfactory nerve layer**				**Glomerular layer**				**External plexiform layer**				**Central regions^a^**			
	**UBB^+1^**	**MC1**	**CP13**	**6F3D**	**UBB^+1^**	**MC1**	**CP13**	**6F3D**	**UBB^+1^**	**MC1**	**CP13**	**6F3D**	**UBB^+1^**	**MC1**	**CP13**	**6F3D**
**BRAAK 0**																
1	−	−	−	−	−	−	−	−	−	−	−	−	−	−	−	−
**BRAAK 3**																
4	NA	NA	NA	NA	NA	NA	NA	NA	+^*^	+	−	−	++	+++	−	−
**BRAAK 6**																
9	−	−	−	−	+	+	−	−	+^*^	+	−	−	++	+++	−	−
10	−	NA	−	−	+	+	+	−	+	NA	−	−	++	NA	+	+

*Paraffin-embedded tissue of the olfactory bulb, olfactory tract and olfactory cortex*.

a *Presumably comprises mitral cell, internal plexiform, and granule cell layers*.

* *Density of immunoreactivity exclusively refers to the occurrence of NTs, the region is devoid of neuronal staining, such as NFTs*.

*NA, tissue was not available for analysis*.

*−, No ir; +, low ir; ++ moderate ir or +++ high ir based on densities of NFTs, plaques, and positive neuronal cells*.

Staining and analysis were also performed on cryoprotected olfactory tissue sections (Table 1, Figure 3). In these samples, the anatomical subdivisions of the human OB were not always clear to distinguish. In the external part of the bulb, the sections presented a layer recognizable as the olfactory nerve layer. Adjacent regions, most likely the glomerular layer, exhibited rounded structures organized in a line over the surface of the bulb. More internally, a layer composed of fibers, most likely the external plexiform layer was distinguishable. In the central portions of these sections, a high number of cells could be visualized. Presumably, these regions comprised the mitral cell, the internal plexiform, and the granule cell layers. Immunohistochemical expression of UBB^+1^ and AD-specific neuropathology in the OB and olfactory tract (more specifically the AON) is illustrated in Figure 3. UBB^+1^ immunoreactivity was mainly restricted to central portions of the bulb, external layers exhibiting either no pathology or few isolated cells and stained threads. All three AD cases (cryoprotected tissue) expressed low to high density of NTs and NFTs throughout the layers of the bulb and expressed few isolated UBB^+1^-positive cells in the central regions. Only one case (patient #10) showed Aβ deposition, which was restricted to the central regions of the bulb namely the granule cell layer.

**Figure 5:**
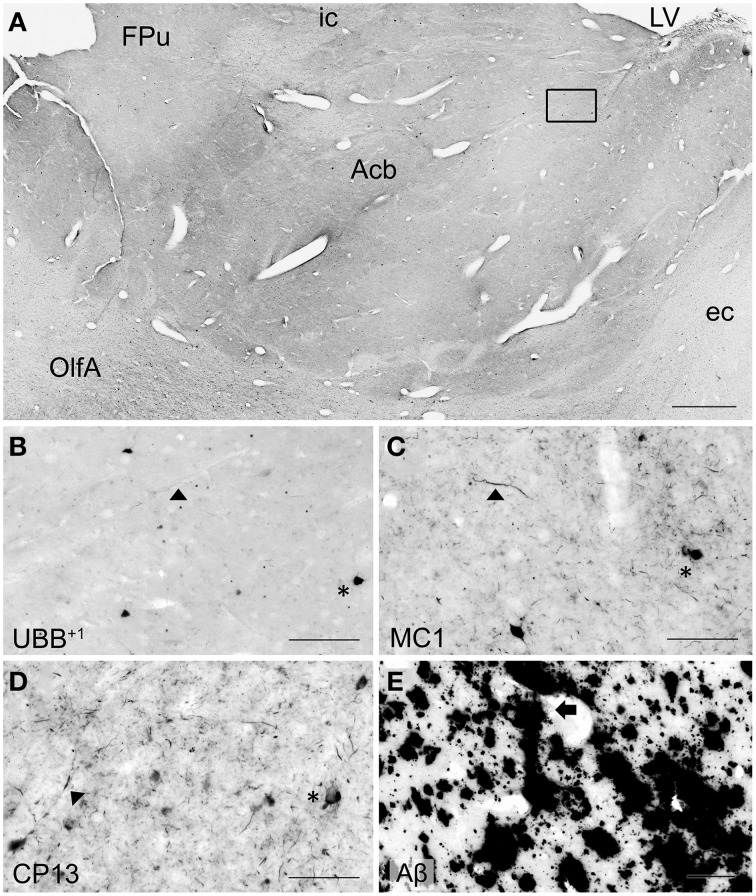
**UBB^+1^ and AD neuropathology (NFTs, NTs, and Aβ plaques) in the human basal ganglia. (A)** Photomicrograph of a 50 μm-thick coronal section from the Acb of patient #5 (Braak 5). **(B)** Higher magnification of the boxed region in **(A)** incubated with the antibody against UBB^+1^, **(C–E)** Higher magnifications of boxed area in adjacent sections stained for **(C)** pre-tangles material (MC1), **(D)** tangles (CP13) and **(E)** Aβ plaques. The Acb, together with other nuclei of the basal ganglia (Cd and Pu), presents little UBB^+1^-immunoreactivity *(asterisk)*. *Asterisks* show neurons with intracellular accumulations. NTs are shown by *filled triangles* and Aβ plaques by an *arrow*. Bars: **(A)**, 1 mm, **(B–E)**, 200 μm. Acb, nucleus accumbens, ec, external capsule; FPu, nucleus accumbens putaminal fundus; ic, internal capsule; LV, lateral ventricle; OlfA, olfactory area.

**Figure 6:**
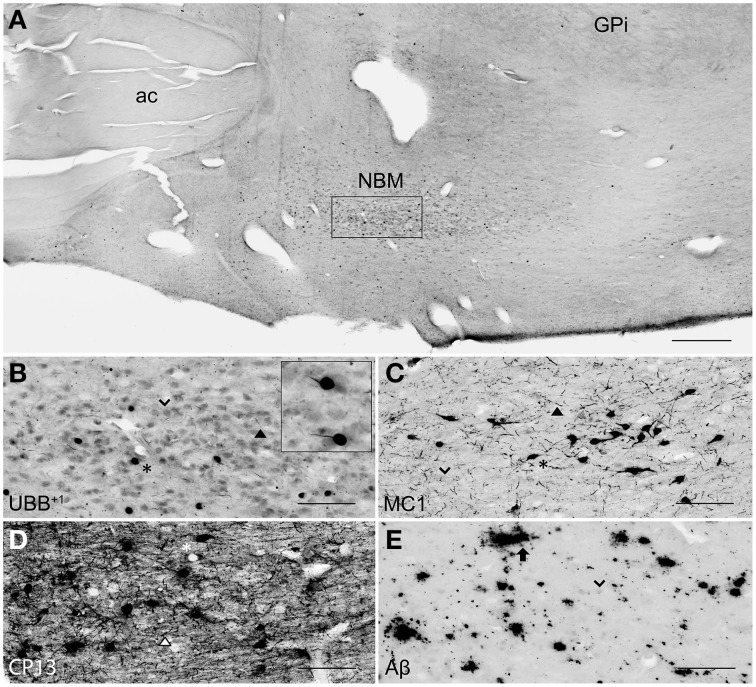
**UBB^+1^ and AD neuropathology (NFTs, NTs, and Aβ plaques) in the human NBM. (A)** Representative photomicrograph of a 50 μm-thick coronal section from the NBM of patient #4 (Braak 3). **(B–E)** Higher magnifications of the boxed region in **(A)** incubated with four antibodies against **(B)** UBB^+1^, **(C)** pre-tangles material (MC1), **(D)** tangles (CP13), and **(E)** Aβ plaques. The cholinergic cells of the NBM are shown by an *arrowhead*. The nucleus is significantly affected by both tau pathology [neuronal staining (*asterisk*) and NTs (*filled triangle*)] and extracellular Aβ accumulation *(arrow)*. UBB^+1^ expression *(asterisk)* is found in cholinergic cells of the NBM. Bars: **(A)**, 1 mm, **(B–E)**, 200 μm. ac, anterior commissure; GPi, globus pallidus internal segment; NBM, nucleus basalis of Meynert.

With respect to the human basal ganglia including the putamen (Pu) and caudate nucleus (Cd) (forming the neostriatum), globus pallidus (GP) and adjacent regions, such as the Acb and the NBM. In all AD cases, AD-related pathology (plaques and NFTs) was present in the basal ganglia (Table 4). The Braak 3 and the four Braak 5 cases exhibited comparable densities of depositions in the basal ganglia nuclei and in the NBM. The non-demented controls displayed occasional NFTs and UBB^+1^-positive cells in the NBM, whereas the nuclei of the basal ganglia of the same cases were not affected. Immunohistochemical expression of UBB^+1^ and AD-specific neuropathology in the Acb and in the NBM is illustrated in Table 4 and in Figures 5, 6.

**Table 4:** **Immunoreactivity for UBB^+1^, pre-tangle material (MC1), tangles (CP13), and plaques (6F3D) in vibratome sections of the basal ganglia of controls (Braak stage 0), intermediate phase (Braak stage 3) and AD patients (Braak stage 5)**.

**Case**	**Nucleus accumbens**				**Striatum (Pu/Cd)**				**Globus pallidus**				**Nucleus basalis of Meynert**			
	**UBB^+1^**	**MC1**	**CP13**	**6F3D**	**UBB^+1^**	**MC1**	**CP13**	**6F3D**	**UBB^+1^**	**MC1**	**CP13**	**6F3D**	**UBB^+1^**	**MC1**	**CP13**	**6F3D**
**BRAAK 0**																
2	–	–	–	–	–	–	–	–	–	–	–	–	+	+	+	–
3	–	–	–	–	–	–	–	–	–	–	–	–	+	–	+	–
**BRAAK 3**																
4	−	+	++	+++	–	–	–	++	–	–	–	–	–	+++	+++	++
**BRAAK 5**																
5	+^*^	++	++	+++	+	+	+	+++	–	–	–	–	–	+++	+++	+++
6	–	–	+	+++	+	+	+	+++	–	–	–	–	++	++	+++	+++
7	–	–	+	+++	+	+	+	+++	–	–	–	–	NA	NA	NA	NA
8	–	–	+	+++	+	+	+	+++	–	–	–	–	++	++	+++	+++

*NA, tissue was not available for analysis*.

*−, No ir; + low ir, ++ moderate ir, or +++ high ir based on densities of NFTs, plaques and positive neuronal cells*.

* *Expression of UBB^+1^ in the Acb of one patient (#5) which was also affected by tau pathology*.

There was a differential distribution of AD-related pathology within several structures. In the putamen, a rostro-caudal gradient could be detected with respect to Aβ deposition, meaning that rostral portions of the nucleus exhibited a greater density of plaques than caudal portions. Furthermore, NFTs were homogeneously distributed in the rostral Pu, whereas the caudal Pu displayed higher concentrations of tau pathology in the ventral area (PuV) and in the regions adjacent to lateral medullary lamina (lml), compared to central portions. The Cd expressed a homogeneous distribution of plaques, and a concentration of occasional NFTs adjacent to the internal capsule (ic). Comparable with the Cd, the Acb displayed homogenous densities of plaques and occasional NFTs among its subdivisions: subventricular (AcSV), centromedial (AcCM), medial (AcM), and centrolateral (AcCL) regions. The caudate fundus (FCd) and putaminal fundus (FPu), together with Acb part of the ventral striatum, showed plaques and ocassionally some NFTs. The internal (GPi) and external (GPe) divisions of the GP exhibited, in a few cases, occasional NFTs and NTs that were restricted to areas adjacent to the medial medullary lamina (mml) and the lml. In contrast, central areas of the nucleus were not affected.

Several structures of the basal ganglia showed selective labeling with either amyloid deposition or tau pathology. Acb, Cd, and Pu expressed severe amyloid pathology, of both the neuritic and non-neuritic type, but showed only few NFTs and NTs. On the other hand, the NBM was significantly affected by both amyloid depositions and tau pathology. GPe and GPi did not exhibit plaques, and were only rarely affected by NFTs and NTs.

UBB^+1^ immunoreactivity was not found in the GP. But the Cd and Pu did express low densities of UBB^+1^ in regions also affected by tau pathology, including FCd, FPu, PuV, and the areas along lml and ic. UBB^+1^ was present in low levels in the Acb of only one Braak 5 patient (#5) which was also affected by tau pathology. The NBM exhibited a moderate density of immunoreactivity, which appeared to selectively target cholinergic cells of the nucleus.

#### The inferior colliculus and raphe nuclei of the human brain

In the human tissue, plaques, NFTs and UBB^+1^-immunoreactive cells were present in the brainstem nuclei of all AD cases (Table 5, Irmler et al., 2012). These nuclei presented a differential distribution of pathology among their subdivisions.

**Table 5:** **Immunoreactivity for UBB^+1^, pre-tangle material (MC1), tangles (CP13), and plaques (6F3D) in vibratome sections of the IC and the raphe nuclei of controls (Braak stage 0), intermediate phase (Braak stage 3) and AD patients (Braak stage 5)**.

**Case**	**Inferior colliculus**				**Dorsal raphe nucleus**				**Median raphe nucleus**			
	**UBB^+1^**	**MC1**	**CP13**	**6F3D**	**UBB^+1^**	**MC1**	**CP13**	**6F3D**	**UBB^+1^**	**MC1**	**CP13**	**6F3D**
**BRAAK 0**												
2	NA	NA	NA	NA	−	−	−	−	−	−	−	−
3	−	−	+	−	−	−	+	−	−	−	+	−
**BRAAK 3**												
4	−	+	+	+++	+	++	+++	+	−	−	+	−
**BRAAK 5**												
5	−	+	+	+++	++	+++	+++	++	++	+++	+++	++
6	−	+++^*^	+++^*^	+++	+	++	+++	−	+	++	+++	−
7	NA	NA	NA	NA	+	+++	++	−	++	++	+++	−
8	−	+	+	+++	++	+++	+++	−	++	+++	+++	+

* *Immunoreactivity is almost exclusively related to the presence of neuritic plaques; the region expresses only few NFTs*.

*NA, tissue was not available for analysis*.

*−, No ir; + low ir, ++ moderate ir, or +++ high ir based on densities of NFTs, plaques and positive neuronal cells*.

**Figure 7:**
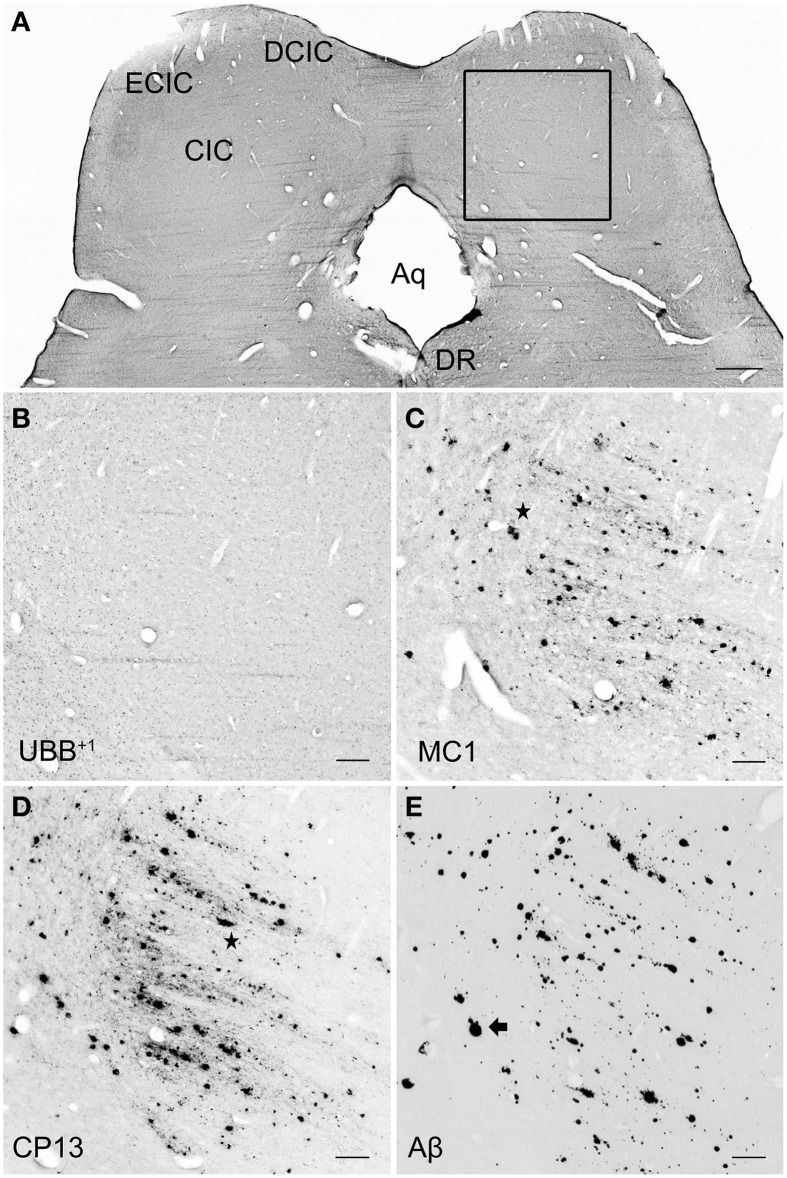
**UBB^+1^ and AD neuropathology (NFTs, NTs, and Aβ plaques) in the human IC. (A)** Representative photomicrograph of a 50 μm-thick coronal section from the brainstem of patient #6 (Braak 5). **(B–E)** Higher magnifications of the boxed region in **(A)** incubated with four antibodies against **(B)** UBB^+1^, **(C)** pre-tangles material (MC1), **(D)** tangles (CP13), and **(E)** Aβ plaques. The IC show selective vulnerability to Aβ accumulation, expressing a high number of both neuritic *(star)* and non-neuritic *(arrow)* plaques. Neuronal staining, represented by NFTs and UBB^+1^-immunoreactive substrates, is not present in this region. Bars: **(A)**, 1 mm, **(B–I)**, 200 μm. Aq, cerebral aqueduct; CIC, central nucleus of IC; DCIC, dorsal cortex of IC; DR, dorsal raphe nucleus; ECIC, external cortex of IC; IC, inferior colliculus.

In the IC, specifically the CIC was severely affected with plaques in 100% of AD cases, independent of Braak stage. By contrast, the DCIC and the ECIC subnuclei were relatively spared. The inferior colliculi showed selective vulnerability to Aβ accumulation, expressing a high number of both neuritic and non-neuritic plaques (Table 5, Figure 7). However, neuronal expression of (pre)-tangles and UBB^+1^-immunoreactive substrates was not present in these regions (Table 5, Figure 7).

The human raphe tissue showed that the non-demented controls exhibited either no pathology or few NFTs and NTs and did not exhibit plaques or UBB^+1^-positive cells. The Braak 3 patient expressed less pathology than the four Braak 5 patients, particularly in the MnR. The MnR and DR express selective vulnerability to tau pathology, being almost devoid of plaques. Both MnR and DR were strongly stained by antibodies against pre-tangles (MC1) and NFTs (CP13) and presented a moderate number of UBB^+1^-positive structures.

**Figure 8:**
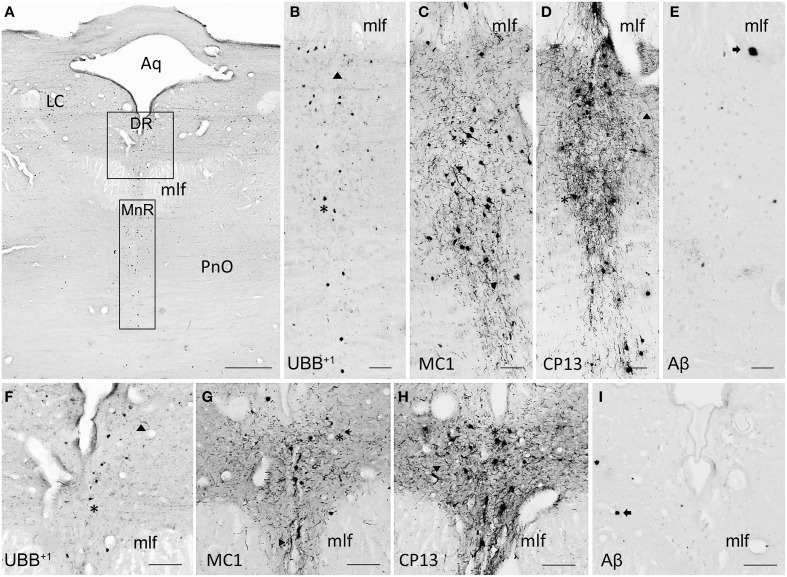
**UBB^+1^ and AD neuropathology (NFTs, NTs, and Aβ plaques) in the human raphe nuclei. (A)** Photomicrograph of a 50 μm-thick section from the brainstem cut perpendicular to the long axis of the spinal cord of patient #8 (Braak 5). **(B–E)** Higher magnifications of the MnR [lower boxed region in **(A)**] incubated with four antibodies against **(B)** UBB^+1^, **(C)** pre-tangles material (MC1), **(D)** tangles (CP13), and **(E)** Aβ plaques. **(F–I)** Larger magnifications of the DR [upper boxed region in (a)] incubated with the same four antibodies. **(F)** Ubi2A; **(G)** MC1; **(H)** CP13; **(I)** 6F3D. The raphe nuclei located in the rostral brainstem (MnR and DR) express selective vulnerability to tau pathology (*asterisk*, neuronal staining; *filled triangle*, NTs), being almost devoid of plaques *(arrow*). Both MnR and DR are strongly stained by antibodies directed against pre-tangle material (MC1) and NFTs (CP13), and present a moderate number of UBB^+1^-positive substrates *(asterisk)*. Bars: **(A)**, 1 mm, **(B–I)**, 200 μm. Aq, cerebral aqueduct; DR, dorsal raphe nucleus; LC, locus coeruleus; mlf, medal longitudinal fasciculus; MnR, median raphe nucleus, PnO, pontine reticular nucleus oral part.

The subdivisions of the DR, namely dorsal DRD, DRV, DRVL, and DRI were equally affected by AD-related pathology with a moderate number of UBB^+1^ positive structures and high number of tangles. In the MnR, immunoreactive substrates, such as NFTs and NTs, were especially concentrated in the medial division (mMnR), while the PMnR exhibited AD-related pathology to a lesser extent (only expressing NTs, but not NFTs). Results are summarized in Table 5 and Figure 8.

## Discussion

The present study describes an extensive topographic mapping of the distribution of UBB^+1^ in the brain of 3413 mice using a sensitive (i.e., ABC technique) immunohistochemical method (Fischer et al., 2009; Irmler et al., 2012). As a next step, the significance of UBB^+1^ and neuropathological markers in five brain areas in the mouse brain was compared with the distribution in human postmortem AD brain tissue. Currently, it is not known in either mouse or human whether the distribution of UBB^+1^ indicates that UBB^+1^ affects the phenotype everywhere it is present. However, neuropathological studies provide possible links with several additional brain regions, AD phenotype and/or proteasomal dysfunction as discussed below. This was already described in our earlier study (Irmler et al., 2012) for the brainstem region where UBB^+1^ was found in respiratory centers in the mouse brain which could also be translated to the human brain. Although the value of transgenic models must not be overestimated, our anatomical and functional study in the brainstem of line 3413 showed predictive power for AD (Irmler et al., 2012). Therefore, the presence of UBB^+1^ can be a useful indicator in other neurodegenerative diseases as shown recently for ER-associated degradation (ERAD) dysfunction in familial encephalopathy with neuroserpin inclusion bodies (FENIB) (Schipanski et al., 2014).

In the next paragraphs, we will discuss the staining results in mouse and human tissue per region, the comparison between the two species and possible links with other neuropathological studies.

### Olfactory bulb and olfactory cortex

Aging is associated with a decrement in olfactory function, represented by a progressive decline in the ability to detect, identify, and discriminate odors (Mobley et al., 2014). Olfactory deficits have been also described in a variety of neurodegenerative disorders, including AD (Attems et al., 2014). Dysfunction in olfaction is a predictor of the incidence of mild cognitive impairment (MCI) and of the conversion of MCI to AD (Djordjevic et al., 2008).

As mentioned above, the UBB^+1^ tg mouse line 3413 accumulates aberrant ubiquitin in low to moderate densities in the OB and the AOB. In contrast, in humans the olfactory structure, which lacks AOB, is relatively spared. Thus, UBB^+1^ seems not to be involved in functional impairment of neural cells at lower levels of the processing pathway. UBB^+1^-positive cells, however, were known to accumulate in areas (e.g., hippocampus and frontal cortex) that are targeted by the neurons of the primary olfactory cortex (Fischer et al., 2009). Therefore, it can be speculated that occurrence of UBB^+1^− positive cells might rather be implicated in the emergence of symptoms associated with higher-order processing, such as inability to identify or discriminate among odors.

Another focus point is the possible association between noradrenergic deficiency and olfactory dysfunction in AD. Tg2576 mice showed olfactory dysfunction together with degeneration of noradrenergic neurons in the LC, the main area of noradrenaline production (Guerin et al., 2009) and projections to the OB. Additionally, LC degeneration in APP23 tg mice enhances inflammation, amyloid plaque load, and neuronal cell death in LC projection regions (e.g., OB) resulting in reduced neuronal integrity and cognitive performance (Heneka et al., 2006). Furthermore, it was demonstrated in an AD mouse model (APPswe, PSEN1dE9) mice that induction of LC degeneration induced exacerbation of olfactory short term memory deficits, a decline in olfactory discrimination and an increase in Aβ load in the granule cell layer of the OB (Rey et al., 2012). The LC is one of the brainstem nuclei wherein tau pathology is apparent, prior to the occurrence of cortical β-amyloid pathology (Braak et al., 2011). Notably, UBB^+1^-immunoreactivy was also present in the LC (Irmler et al., 2012). Consequently, UBB^+1^ accumulation might be involved in the noradrenergic neurodegeneration in the LC and olfactory dysfunction in AD. Therefore, performing odor tests with line 3413 would provide valuable information to the question whether UBB^+1^ accumulation in OB and LC is reflected in an impaired olfactory dysfunction. UBB^+1^ in the AOB could be associated with and could be tested for social and reproductive behaviors.

With respect to neuropathology, the present study also investigated the distribution of amyloid β and tau in the human olfactory bulb, tract and primary olfactory cortex of AD patients and controls. The human olfactory cortex expressed both NFTs and Aβ plaques, whereas olfactory bulb and tract more commonly exhibit cytoskeletal pathology. Low densities of NFTs and NTs were found in external layers of the bulb, namely glomerular and external plexiform layers. Higher densities were instead detected in central areas comprising mitral cell, internal plexiform and granule cell layers. In the olfactory tract immunoreactivity was restricted to the AON. Consistent with the results of the present study, Braaks group found accumulation of NFTs, NTs, and plaques in this nucleus (Ohm and Braak, 1987).

In conclusion, the olfactory region is relatively spared in humans for UBB^+1^ expression and therefore there is no one to one relationship between mouse and human for this region. The present study confirmed the presence of plaques and tangles in the human olfactory region.

### Basal ganglia

The different subdivisions of the Acb, involved in reward and reinforcement mechanisms and in regulating emotional behavior (De Jong et al., 2011), showed in the 3413 tg line a high density of UBB^+1^ immunoreactive cells and a moderate UBB^+1^ cell-intensity.

By contrast, in the human brain UBB^+1^ was present only in few isolated cells of Acb in a single AD case. In all AD patients, Acb showed severe Aβ pathology in both neuritic and non-neuritic plaques, however only a few NFTs and NTs were present. Comparably, senile plaques were demonstrated in the ventral striatum of AD patients (Suenaga et al., 1990). A number of studies have shown dopaminergic (DAergic) dysfunction in AD patients, mainly in the striatum. As the DAergic brain reward system is essential to experience motivation and pleasure, DAergic dysfunction in AD patients was well-correlated with apathy defined as a lack of motivation. Apathetic symptoms are detected in up to 47% of patients with mild AD and up to 80% in those with severe AD (Mitchell et al., 2011). A reduction in dopamine uptake was shown in the Acb in AD patients compared to non-demented controls (Murray et al., 1995).

These data suggest that there is no relation between mouse and human on UBB^+1^ expression in the Acb. Apparently, proteasomal malfunctioning seems not to be involved in the DAergic dysfunction seen in AD.

Additionally to the Acb, the mouse Pu and Cd are more affected by UBB^+1^ accumulation compared to the human brain while the GP was spared of any UBB^+1^ labeling for both mouse and human. We also demonstrated that the human Pu and Cd were severely affected by amyloid deposition and being almost devoid of cytoskeletal aggregates, whereas GP was relatively spared of any pathology. This is consistent with previous work demonstrating diffuse plaques in Cd, Acb, and Pu combined with an absence of plaques in GP (Brilliant et al., 1997). Taken together, these data suggest that NFTs, as well as UBB^+1^, seem not to be involved in the pathological changes that affect this part of the human basal forebrain. However, we confirmed the presence of plaques in basal ganglia.

The analysis of the human basal ganglia availed an examination of structures that lie adjacent to them. The NBM is considered one of the structures most susceptible to neurofibrillary degeneration, (Mesulam et al., 2004). Atrophy was shown via MRI in the NBM of AD patients, most pronounced in the posterior subdivision (Ch4p) as well as a reduction in the number of Ch4p cholinergic neurons ranging about 70% of age-matched controls (Jellinger, 2014). The present study demonstrated severe accumulation of tangle-bearing neurons in the NBM of all AD cases, independently of disease progression. The occurrence of few isolated NFTs could be also detected in brains of non-demented controls, which is in line with the finding that cholinergic neurons express neurofibrillary pathology even during normal aging, but increases significantly in MCI and worsens in AD (Mesulam, 2013). A clear presence of UBB^+1^ was also shown in the human AD cases, which is consistent with a previous study (Van Leeuwen et al., 2000). The occurrence of early and severe cytopathology of NBM, together with a profound reduction of cholinergeric innervation (Whitehouse et al., 1982), may be partly responsible for the existence of a wide range of symptoms AD patients commonly suffer from.

### Inferior colliculus

Age-related auditory deficits are quite common in the elderly. AD patients are known to exhibit structural changes (neuronal loss, primary sensory deafferentation) in the central auditory pathways (Sinha et al., 1993). Central auditory dysfunction (CAD) includes individuals hearing well in a quiet environment but having hearing problems in environments with a lot of background noise (e.g., competing conversations). Studies demonstrated an increased risk of AD in individuals with CAD and suggest that CAD is an early manifestation of AD that occurs before any sign of cognitive decline. Indeed, people suffering from MCI showed CAD to a considerable extent (Idrizbegovic et al., 2011). Thus, auditory dysfunction may not only be a valuable tool for diagnosing AD at an early stage, it may also be predictive for the development of AD. Moreover there is substantial evidence for neurodegeneration such as senile plaques (Ohm and Braak, 1989) and neurofibrillary tangles (Dugger et al., 2011) in the medial geniculate nucleus ventral part (MGV), the CIC, the primary auditory and auditory association cortices of AD patients.

In the present study, expression of UBB^+1^ in line 3413 was found in the IC with a density varying from moderate to high. By contrast, in the human brain, UBB^+1^− immunoreactive substrates were not detected in the IC, which is consistent with the finding that UBB^+1^ co-localizes with tau pathology, such as NFTs and dystrophic neurites, but not with amyloid depositions (Van Leeuwen et al., 1998). In line with other research work (Parvizi et al., 2001), the human immunohistochemical experiments demonstrated that CIC is severely affected in AD, contrary to the external layers of the nucleus (ECIC, DCIC), which expressed very low densities, if any, of immunoreactive substrates (plaques or tangles). Furthermore, the IC expressed selective vulnerability to amyloid pathology, being almost devoid of NFTs. One Braak 5 patient (#6) showed a high immunoreactivity for (pre)-tangles, however it is related to its presence in neuritic plaques.

In conclusion, our experiments demonstrated that high concentrations of AD-related pathology (i.e., MC1, CP13, and Aβ) affect the inferior colliculi, especially the CIC, in humans. Furthermore, because the Braak 3 case already exhibits severely amyloid depositions, it can be claimed that pathological changes in the nuclei may initiate early in disease's progression. The involvement of the IC, represented by accumulation of plaques, as well as neuronal loss and altered dendrite arborization (Baloyannis et al., 2009), might, therefore, be partly responsible for central auditory symptoms commonly diagnosed in AD patients.

### Raphe nuclei

Depression is a common comorbidity in individuals with AD and may also precede the clinical symptoms of AD by several years (Sierksma et al., 2010). However, it is unclear whether depression is a risk factor (Geerlings et al., 2008) or a prodromal sign (Wilson et al., 2004) for dementia and AD. One of the possible explanations about the pathophysiology of depression is the “monoaminergic hypothesis” in which a depletion in monoamine levels in the brain i.e., serotonin (5-HT) and noradrenaline (NA) is thought to play a role (Sierksma et al., 2010).

Our human data demonstrated that both DR and MnR accumulate UBB^+1^− positive cells. These results are in line with the finding that aberrant ubiquitin is expressed in comparable brain regions of the 3413 tg mouse. UBB^+1^-ir cells showed a low to moderate density of labeling in certain raphe subnuclei of the 3413 tg mice. Intriguingly, the DR receives afferents from the LC. Noradrenergic as well as serotonergic degeneration of the LC is associated with depression (Ressler and Nemeroff, 2000). It was shown in tg mice that LC degeneration causing NA deficiency in AD contributes to early cognitive deficits (Hammerschmidt et al., 2013). As UBB^+1^-ir cells were shown in both the DR and LC of 3413 tg mice, it is possible that UBB^+1^ accumulation is associated with depression in AD. These results show that there is a correlation with UBB^+1^ expression in the DR and the MnR in line 3413. Because accumulation of UBB^+1^ inhibits proteasomal activity (Van Tijn et al., 2007) and because its occurrence in other brain regions is associated with neuropsychological (Fischer et al., 2009) and behavioral (Irmler et al., 2012) phenotypes, it is likely that the presence of UBB^+1^-positive cells in the raphe nuclei contribute to the abnormal functioning of the 5-HTergic system in AD, as well as to the affective symptoms commonly diagnosed in AD patients.

Additionally, the human data showed that nuclei of the rostral raphe complex, namely DR and MnR are severely affected by NFTs. The caudal raphe complex (RMg, RPa, ROb) was not analyzed, but several studies have already shown that such nuclei are relatively spared in AD (Rub et al., 2000). Consistent with other work (Parvizi et al., 2001), we demonstrated that the DR and MnR are selectively vulnerable to cytoskeletal pathology, being almost devoid of plaques. In addition, it was found that AD-related pathology in the raphe complex correlates with disease progression (Rub et al., 2000). The DR manifests lesions at early stages (Braak 1-2), whereas MnR initiates to show pathology later (Braak 3-4). Congruently, the Braak 3 patient analyzed in the present study showed NFTs in DR, but not in MnR. It has been proposed, that neuropathological changes, such as the aggregation of hyperphosphorylated tau, begin in the raphe nuclei, subsequently spreading to the transentorhinal cortex apparently via seeding (Grinberg et al., 2009; Jucker and Walker, 2011). Accordingly, 20% of Braak 0 cases and 100% of Braak ≥ 1 expressed NFTs in the DR (Grinberg et al., 2009). Our findings are consistent with this hypothesis.

## Concluding remarks

The present study expands prior work and shows the global UBB^+1^ distribution in the brain of the tg mouse line 3413. We selected five regions, namely the OB, Acb, NBM, IC, and raphe nuclei. The results support the idea that AD is a phenomenon that involves more than forebrain degeneration associated with memory problems. It has been shown that the 3413 tg model has some predictive value, as also noted previously (Irmler et al., 2012). However, because it is a genetic model that is useful to address loss of protein control, it has limitations with respect to AD which is a multifactorial disease. Line 3413 can be used as a read-out possibility for further AD-related research in mice especially when crossed with existing AD lines and other diseases models to show the relevance of other processes such as Aβ plaque formation (Van Tijn et al., 2012) and other cytological processes (Schipanski et al., 2014). The present study showed that immunoreactivity for UBB^+1^ found in the tg mouse model 3413 is not completely mirrored in the AD brain. More specifically, we demonstrated in addition to the brain stem nuclei that areas that present substantial accumulation of tangle bearing neurons, such as NBM and raphei nuclei, present also high densities of UBB^+1^-positive cells. These data can be used to reveal the impact of proteasomal stress on functioning and on neurodegenerative pathology as shown by GWAS studies in AD (International Genomics of Alzheimer's Disease Consortium (IGAP), 2015) and to uncover new avenues for research not only on AD but also on other multifactorial tauopathies (Fischer et al., 2003) and polyglutamine diseases (such as HD, De Pril et al., 2004).

## Ethical standard

Animals were handled according to local ethical guidelines.

### Conflict of interest statement

The authors declare that the research was conducted in the absence of any commercial or financial relationships that could be construed as a potential conflict of interest.

## List of anatomical abbreviations used in the paper

**Table:**

**Abbreviations**	
10N	Dorsal motor nucleus of vagus
AAD	Anterior amygdaloid area, dorsal part
AAV	Anterior amygdaloid area, ventral part
Ac	Nucleus of the anterior commissure
Acb	Accumbens nucleus
AcbC	Accumbens nucleus, core
AcbSh	Accumbens nucleus, shell
AcCL	Subventricular part of n. accumbens
AcCM	Centromedial part of n. accumbens
AcM	Medial part of n. accumbens
Aco	Anterior cortical amygdaloid nucleus
AcSV	Centrolateral part of n. accumbens
Ahi	Amygdalohippocampal area
AhiAL	Amygdalohippocampal area, anterolateral part
AhiPM	Amygdalohippocampal area, posteromedial part
AOB	Accessory olfactory bulb
AOD	Anterior olfactory area, dorsal part
AOE	Anterior olfactory area, external part
AOL	Anterior olfactory area, lateral part
AOM	Anterior olfactory area, medial part
AON	Anterior olfactory nucleus
AOP	Anterior olfactory area, posterior part
AOV	Anterior olfactory area, ventral part
AP	Area postrema
Apir	Amygdalopiriform transition area
Aq	Cerebral aqueduct
Astr	Amygdalostriatal transition area
BAOT	Bed nucleus of the accessory olfactory tract
BIC	Nucleus of the brachium of the inferior colliculus
BLA	Basolateral amygdaloid nucleus, anterior part
BLP	Basolateral amygdaloid nucleus, posterior part
BLV	Basolateral amygdaloid nucleus, ventral part
BMA	Basomedial amygdaloid nucleus, anterior part
BMP	Basomedial amygdaloid nucleus, posterior part
BST	Bed nucleus of the stria terminalis
CA1	Cornu ammonis 1 (CA1) of hippocampus
CA2	Cornu ammonis 2 (CA2) of hippocampus
CA3	Cornu ammonis 3 (CA3) of hippocampus
Cb	Cerebellum
Cd	Caudate nucleus
CeC	Central amygdaloid nucleus, capsular part
CeL	Central amygdaloid nucleus, lateral division
CeM	Central amygdaloid nucleus, medial division
CeMAD	Central amygdaloid nucleus, medial division, anterodorsal part
CeMAV	Central amygdaloid nucleus, medial division, anteroventral part
CeMPV	Central amygdaloid nucleus, medial posteroventral part
CEnt	Caudomedial entothinal cortex
Cg1	Cingulate cortex, area 1
Cg2	Cingulate cortex, area 2
CIC	Central nucleus of the inferior colliculus
Cli	Caudal linear nucleus of the raphe
CPu	Caudate putamen (striatum)
Cx	Cerebral cortex
CxA	Cortex-amygdala transition zone
DC	Dorsal cochlear nucleus
DCIC	Dorsal cortex of the inferior colliculus
Den	Dorsal endopiriform nucleus
DP	Dorsal peduncular cortex
DR	Dorsal raphe nucleus
DRC	Dorsal raphe nucleus, caudal part
DRD	Dorsal raphe nucleus, dorsal part
DRI	Dorsal raphe nucleus, interfascicular part
DRV	Dorsal raphe nucleus, ventral part
DRVL	Dorsal raphe nucleus, ventrolateral part
DTT	Dorsal tenia tecta
Ec	External capsule
ECIC	External cortex of the inferior colliculus
Ect	Ectorhinal cortex
E/OV	Ependymal and subendymal layer/olfactory ventricle
Epl	External plexiform layer of the olfactory bulb
EplA	External plexiform layer of the accessory olfactory bulb
FC	Fasciola cinereum
FCd	Nucleus accumbens, caudate fundus
Fpu	Nucleus accumbens, putaminal fundus
FrA	Frontal association cortex
Gi	Gigantocellular reticular nucleus
Gl	Glomerular layer of the olfactory bulb
GlA	Glomerular layer of the accessory olfactory bulb
GP	Globus pallidus
Gpe	Globus pallidus, external segment
Gpi	Globus pallidus, internal segment
GrA	Granule cell layer of the accessory olfactory bulb
GrC	Granular layer of the cochlear nuclei
GrDG	Granular layer of the dentate gyrus
GrO	Granular cell layer of the olfactory bulb
Hip	Hippocampus
HTh	Hypothalamus
I	Intercalated nuclei of the amygdala
Ic	Internal capsule
IC	Inferior colliculus
ICj	Islands of Calleja
ICjM	Islands of Calleja, major island
Ig	Indusium griseum
IL	Infralimbic cortex
IM	Intercalated amygdaloid nucleus, main part
IPl	Internal plexiform layer of the olfactory bulb
L1	Cortical layer 1
L2	Cortical layer 2
L3	Cortical layer 3
L4	Cortical layer 4
L5a	Cortical layer 5a
L5b	Cortical layer 5b
L6	Cortical layer 6
La	Lateral amygdaloid nucleus
LAcbSh	Lateral accumbens shell
LaDL	Lateral amygdaloid nucleus, dorsolateral part
LaVL	Lateral amygdaloid nucleus, ventromedial part
LaVM	Lateral amygdaloid nucleus, ventromedial part
LC	Locus coeruleus
LGP	Lateral globus pallidus
lml	Lateral medullary lamina
LMol	Lacunosum moleculare layer of the hippocampus
LO	Lateral orbital cortex
LOT	Nucleus of the lateral olfactory tract
LPB	Lateral parabrachial nucleus
LPBC	Lateral parabrachial nucleus, central part
LPBD	Lateral parabrachial nucleus, dorsal part
LPBE	Lateral parabrachial nucleus, external part
LPBI	Lateral parabrachial nucleus, internal part
LPBV	Lateral parabrachial nucleus, ventral part
LS	Lateral septal nucleus
LSO	Lateral superior olive
LSS	Lateral stripe of the striatum
LV	Lateral ventricle
M2	Secondary motor cortex
MeA	Medial amygdaloid nucleus, anterior part
MeAD	Medial amygdaloid nucleus, anterior dorsal part
MeAV	Medial amygdaloid nucleus, anteroventral part
MePD	Medial amygdaloid nucleus, posterodorsal part
MePV	Medial amygdaloid nucleus, posteroventral part
MG	Medial geniculate nucleus
MGD	Medial geniculate nucleus, dorsal part
MGM	Medial geniculate nucleus, medial part
MGP	Medial globus pallidus
MGV	Medial geniculate nucleus, ventral part
Mi	Mitral cell layer of the olfactory bulb
MiA	Mitral cell layer of the accessory olfactory bulb
Mlf	Medial longitudinal fasciculus
Mml	Medial medullary lamina
mMnR	Median raphe nucleus, medial part
MnR	Median raphe nucleus
Mol	Molecular layer of the dendate gyrus
MPB	Medial parabrachial nucleus
MPBe	Medial parabrachial nucleus external part
MZMG	Marginal zone of the medial geniculate
NBM	Nucleus basalis of Meynert
NCS	Nucleus centralis superior
OB	Olfactory bulb
OlfA	Olfactory area
PAG	Periaqueductal gray
PaS	Parasubiculum
PBW	Parabrachial nucleus waist part
Pir	Piriform cortex
PLCo	Posterolateral cortical amygdaloid nucleus (C2)
PMCo	Posteromedial cortical amygdaloid nucleus (C3)
PMnR	Paramedian raphe nucleus
PnC	Pontine reticular nucleus, caudal part
PnO	Pontine reticular nucleus, oral part
PnR	Pontine raphe nucleus
PoDG	Polymorph layer of the dentate gyrus
PrL	Prelimbic cortex
PrS	Presubiculum
PSol	Parasolitary nucleus
Pu	Putamen
PuV	Ventral area of putamen
Py	Pyramidal cell layer of the hippocampus
Rad	Stratum radiatum of the hippocampus
RC	Raphe cap
RLi	Rostral linear nucleus of the raphe
RMg	Raphe magnus nucleus
ROb	Raphe obscurus nucleus
RPa	Raphe pallidus nucleus
RSD	Retrosplenial dysgranular cortex
RSGa	Retrosplenial granular cortex, a part
RSGb	Retrosplenial granular cortex, b part
RSGc	Retrosplenial granular cortex, c part
S	Septal area
Sb	Subiculum
SC	Superior colliculus
Shi	Septohippocampal nucleus
SLEA	Sublenticular extended amygdala
SLEAC	Sublenticular extended amygdala, central part
SLEAM	Sublenticular extended amygdala, medial part
Slu	Stratum lucidum, hippocampus
Sol	Nucleus of the solitary tract
Sol	Solitary tract
SolC	Nucleus of the solitary tract, commissural part
SolCe	Nucleus of the solitary tract, central part
SolDL	Nucleus of the solitary tract, dorsolateral part
SolDM	Nucleus of the solitary tract, dorsomedial part
SolG	Nucleus of the solitary tract, gelatinous part
SolI	Nucleus of the solitary tract, interstitial part
SolIM	Nucleus of the solitary tract, intermediate part
SolM	Nucleus of the solitary tract, medial part
SolV	Nucleus of the solitary tract, ventral part
SolVL	Nucleus of the solitary tract, ventrolateral part
STh	Subthalamic nucleus
Th	Thalamus
Tu	Olfactory tubercle
Tz	Nucleus of the trapezoid body
V1	Primary visual cortex
V2ML	Secondary visual cortex, mediolateral area
V2MM	Secondary visual cortex, mediomedial area
VC	Ventral cochlear nucleus
VCA	Ventral cochlear nucleus, anterior part
VCP	Ventral cochlear nucleus, posterior part
VEn	Ventral endopiriform nucleus
VO	Ventral orbital cortex
VTT	Ventral tenia tecta

## Abbreviations

Aβ: amyloid β protein
ABC: avidin-biotin complex
AD: Alzheimer's disease
APOE: apolipoprotein E
APP: amyloid precursor protein
CAD: central auditory dysfunction
CaMKIIα: Calmodulin kinase II alpha
D: density
DAB: diaminobenzidine
DAergic: dopaminergic
DUB: deubiquitinating enzyme
FENIB: familial encephalopathy with neuroserpin inclusion bodies
ERAD: ER-associated degradation
GWAS: Genome Wide Association Studies
HD: Huntington's disease
HTh: hypothalamus
I: intensity
ir: immunoreactivity
MCI: mild cognitive impairment
MRI: magnetic resonance imaging
NA: noradrenaline
NFT: neurofibrillary tangle
NMDA: N-methyl-D-aspartate
NT: neuropil threads
PBS: phosphate-buffered saline
PHF: paired helical filament
PQC: protein quality control
PS1: presenilin 1
PS2: presenilin 2
RT: room temperature
SPECT: single-photon emission computed tomography
TBS: Tris-buffered saline
tg: transgenic
TREM2: triggering receptor expressed on myeloid cells 2
UBB: ubiquitin B
UBB^+1,^ ubiquitin B^+1;^ UBB^+1^I: intensity of UBB^+1^ immunoreactivity
UBB^+1^D: density of UBB^+1^ immunoreacitivity
UBC: ubiquitin C
UPS: ubiquitin-proteasome system
5-HT: serotonin.
