# Supplementary material for: Localization of mutant ubiquitin in the brain of a transgenic mouse line with proteasomal inhibition and its validation at specific sites in Alzheimer's disease
Source: Front Neuroanat. 2015 Mar 17;9:26. doi: 10.3389/fnana.2015.00026 (PMC4362318; doi:10.3389/fnana.2015.00026)
Supplement: Supplementary file 1 [file DataSheet1.DOCX]

**SUPPLEMENTS**

**Supplementary results**

## UBB^+1^ in the telencephalon and diencephalon

### Telencephalic structures in the mouse brain

The present study reports other telencephalic regions to be UBB^+1^ immunoreactive. The bed nucleus of the stria terminalis (BST) exhibited a low to moderate UBB^+1^ intensity and density (supplement table S2). However, in the supracapsular part of the BST (BSTS) no immunoreacitivity was present.

### Diencephalic structures in the mouse brain

The subnuclei of the thalamus and the hypothalamus showed a low to moderate UBB^+1^ staining intensity in a modest number of cells (supplement table S2, figure 2), except the tuber cinereum (TC), part of the hypothalamus, which was completely negative for any UBB^+1^ immunoreactivity. The mammillary bodies demonstrate a low to moderate UBB^+1^ cell-intensity and density Additionally, the subnuclei of the preoptic area and the geniculate area contained a comparable amount of UBB^+1^ expression ranging from a low to moderate cell-intensity and density (supplement table S2). The following diencephalic regions showed a low to moderate UBB^+1^ expression: bed nucleus of the anterior commissure (BAC), interstitial nucleus of the posterior limb of the anterior commissure (IPAC), anteroventral periventricular nucleus (AVPe), lateral (LHb) and medial (MHb) habenular nuclei, retroethmoid nucleus (REth), nucleus of the stria medullaris (SM) and the vascular organ of the lamina terminalis (VOLT) (supplement table S2).

## UBB^+1^ in the mesencephalon Mesencephalic structures in the mouse brain

UBB^+1^ was present in low levels in the superior colliculus (SC) subnuclei of the tg mice. Other tectal regions showed a comparable UBB^+1^ expression level: anterior (APT), posterior (PPT) and olivary (OPT) prectectal nucleus, deep mesencephalic nucleus (DpMe), intercollicular nucleus (InCo), nucleus of the optic tract (OT), parabigeminal nucleus (PBG), subcommissural organ (SCO) and subbrachial nucleus (SubB) (supplementary table S2).

More ventrally, several tegmental structures stained positively for UBB^+1^ as well: periaqueductal gray (PAG), substantia nigra (SN), ventral (VTA), dorsal (DTg), microcellular (MiTg), subpedencular (SPTg) and dorsomedial (DMTg) tegmental area, oculomotor nucleus (3N, 3PC), caudal interstitial nucleus of the medial longitudinal fasciculus (Ci), cuneiform nucleus (CnF), dorsal (DT) and medial (MT) terminal nucleus of the accessory optic tract (DT), epimicrocellular nucleus (Emi), Edinger-Westphal nucleus (EW), interfascicular nucleus (IF), interpeduncular nucleus (IP), mesencephalic trigeminal nucleus (Me5), pararubral (PaR), retrorubral (RR) and prerubral (PR) nucleus and precommissural nucleus (PrC) showed a low UBB^+1^ labeling (supplementary table S2). A moderate expression level was seen in the nucleus of Darkschewitsch (Dk), the interstitial nucleus of Cajal (InC), the paratrochlear nucleus (Pa4), the paranigral nucleus (PN), and the retrorubral field (RRF) (supplementary table S2).

## UBB^+1^ in the pons, cerebellum and medulla oblongata

## Pons and cerebellum structures in the mouse brain

A low to moderate density and intensity is present in a wide spectrum of nuclei in the pons: dorsal (DLL) and ventral part (VLL) of the lateral lemniscus, paralemniscal nucleus (PL), periolivary nuclei (DPO, MVPO, RPO, SPO), trigeminal nuclei (I5, Mo5, P5, Pr5DM, Su5), tegmental nuclei (LDTg, LDTgV, PPTG, RTP, VLTg),facial nucleus (7N), A5 cells, Barrington’’s nucleus (Bar), central gray (CGPn), epirubrospinal nucleus (ERS), perifacial zone (P7), pontine nucleus (Pn), pontine reticular nuclei (PnC, PnO, PnV), and the sphenoid nucleus (Sph) (supplementary table S2).

Medulla oblongata in the mouse brain

Additional medullary nuclei were positive for UBB^+1^ (supplementary table S2) with staining varying from low to moderate: cuneate nuclei (Cu, ECu), reticular nuclei (Gi, GiA, IRt, MdD, Md, PCRt, PCRtA), trigimenal nuclei (DMSp5,Ge5, Pa5, Sp5, Sp5C, Sp5I, Sp5ODM, Sp5OVL), vestibular nuclei (MVe, MVeMC, MVePC, SPVe), C1 and C2 cells, dorsal (DPGi) and lateral (LPGi) paragigantocellular nucleus, gracile nucleus (Gr), rhabdoid nucleus (Rbd) and the nucleus of Roller (Ro). However, the supplementary table S2 demonstrates also different medullary regions completely negative for UBB^+1^ (e.g. Amb, Bo, LRt, LVe,PMn, RAmb,…). UBB^+1^ is also present in the inferior olive nucleus but only the medial (IOM) and the principal nucleus (IOPr).

**Supplementary tables:**

Table S1. Abbreviations of anatomical brain structures listed in alphabetical order.

Table S2. Scoring of the UBB^+1^ immunoreactivity in a wide range of subnuclei located in the telencephalon, diencephalon, mesencephalon, pons, medulla oblongata and cerebellum of 3413 transgenic mice (n=14) compared with WT controls (n=9). Relative UBB^+1^ immunoreactivity scores for intensity and density are shown.

- - **sdfsdfsf**
  - **fsdf**
- **P**

| Abbreviations | |
| --- | --- |
| 3N | oculomotor nucleus |
| 3PC | oculomotor nucleus,  parvicellular part |
| 4N | trochlear nucleus |
| 6N | abducens nucleus |
| 7N | facial nucleus |
| 10N | dorsal motor nucleus of vagus |
| 12N | hypoglossal nucleus |
| A1 | A1 noradrenaline cells |
| A2 | A2 noradrenaline cells |
| A5 | A5 noradrenaline cells |
| A7 | A7 noradrenaline cells |
| A12 | A12 dopamine cells |
| A13 | A13 dopamine cells |
| AA | anterior amygdaloid area |
| AAD | anterior amygdaloid area,  dorsal part |
| AAV | anterior amygdaloid area,  ventral part |
| ac | nucleus of the anterior commissure |
| Acb | accumbens nucleus |
| AcbC | accumbens nucleus, core |
| AcbSh | accumbens nucleus, shell |
| AcCL | subventricular part of n. accumbens |
| AcCM | centromedial part of n. accumbens |
| AcM | medial part of n. accumbens |
| ACo | anterior cortical amygdaloid nucleus |
| AcSV | centrolateral part of n. accumbens |
| AD | anterodorsal thalamic nucleus |
| ADP | anterodorsal preoptic nucleus |
| AHA | anterior hypothalamic area,  anterior part |
| AHC | anterior hypothalamic area,  central part |
| AHi | amygdalohippocampal area |
| AHiAL | amygdalohippocampal area, anterolateral part |
| AHiPM | amygdalohippocampal area, posteromedial part |
| AHP | anterior hypothalamic area, posterior part |
| al | ansa lenticularis |
| AM | anteromedial thalamic nucleus |
| Amb | ambiguus nucleus |
| AMV | anteromedial thalamic nucleus, ventral part |
| Ang | angular thalamic nucleus |
| AO | anterior olfactory area |
| AOB | accessory olfactory bulb |
| AOD | **anterior olfactory area,  dorsal part** |
| AOE | **anterior olfactory area,  external part** |
| AOL | **anterior olfactory area,  lateral part** |
| AOM | **anterior olfactory area,  medial part** |
| AON | anterior olfactory nucleus |
| AOP | **anterior olfactory area,  posterior part** |
| AOV | **anterior olfactory area,  ventral part** |
| AP | area postrema |
| APir | amygdalopiriform transition area |
| APT | anterior pretectal nucleus |
| APTD | anterior pretectal nucleus,  dorsal part |
| APTV | anterior prectectal nucleus,  ventral part |
| Aq | cerebral aquaduct |
| Arc | arcuate hypothalamic nucleus |
| ArcD | arcuate hypothalamic nucleus, dorsalpart |
| ArcL | arcuate hypothalamic nucleus, lateral part |
| ArcLP | arcuate hypothalamic nucleus, lateroposterior part |
| ArcMP | arcuate hypothalamic nucleus, medial posterior part |
| Asc5 | accessory trigeminal nucleus |
| Asc6/7 | accessory abducens nucleus & facial nucleus |
| Asc7 | accessory facial nucleus |
| AStr | amygdalostriatal transition area |
| ATg | anterior tegmental nucleus |
| AV | anteroventral thalamic nucleus |
| AVDM | anteroventral thalamic nucleus, dorsomedial part |
| AVPe | anteroventral periventricular nucleus |
| AVVL | anteroventral thalamic nucleus, ventrolateral part |
| B9 | B9 serotonin cells |
| B4 | B4 serotonin cells |
| BAC | bed nucleus of the anterior commissure |
| BAOT | bed nucleus of the accessory olfactory tract |
| Bar | Barrington's nucleus |
| BIC | nucleus of the brachium of the inferior colliculus |
| BLA | basolateral amygdaloid nucleus, anterior part |
| BLP | basolateral amygdaloid nucleus, posterior part |
| BLV | basolateral amygdaloid nucleus, ventral part |
| BMA | basomedial amygdaloid nucleus, anterior part |
| BMP | basomedial amygdaloid nucleus, posterior part |
| Bo | Botzinger complex |
| BST | bed nucleus of the stria terminalis |
| BSTIA | bed nucleus of the stria terminalis, intraamygdaloid division |
| BSTL | bed nucleus of the stria terminalis, lateral division |
| BSTLD | bed nucleus of the stria terminalis, lateral division, dorsal part |
| BSTLI | bed nucleus of the stria terminalis, lateral division, intermediate part |
| BSTLJ | bed nucleus of the stria terminalis, lateral division, juxtacapsular part |
| BSTLP | bed nucleus of the stria terminalis, lateral division, posterior part |
| BSTLV | bed nucleus of the stria terminalis, lateral division, ventral part |
| BSTMA | bed nucleus of the stria terminalis, medial division, anterior part |
| BSTMP | bed nucleus of the stria terminalis, medial division, posterior part |
| BSTMPI | bed nucleus of the stria terminalis, medial division, posterointermediate part |
| BSTMPL | bed nucleus of the stria terminalis, medial division, posterolateral part |
| BSTMPM | bed nucleus of the stria terminalis, medial division, posteromedial part |
| BSTMPV | bed nucleus of the stria terminalis, medial division, ventral part |
| BSTS | bed nucleus of the stria terminalis, supracapsular part |
| C1 | C1 adrenaline cells |
| C2 | C2 adrenaline cells |
| C3 | C3 adrenaline cells |
| CA1 | cornu ammonis 1 (CA1) of hippocampus |
| CA2 | cornu ammonis 2 (CA2) of hippocampus |
| CA3 | cornu ammonis 3 (CA3) of hippocampus |
| Cb | cerebellum |
| Cd | caudate nucleus |
| CeC | central amygdaloid nucleus, capsular part |
| CeCv | central cervical nucleus |
| CeL | central amygdaloid nucleus,  lateral division |
| CeM | central amygdaloid nucleus,  medial division |
| CeMAD | central amygdaloid nucleus,  medial division, anterodorsal part |
| CeMAV | central amygdaloid nucleus,  medial division, anteroventral part |
| CeMPV | central amygdaloid nucleus,  medial posteroventral part |
| CEnt | caudomedial entothinal cortex |
| CG | central gray |
| Cg1 | cingulate cortex, area 1 |
| Cg2 | cingulate cortex, area 2 |
| CGA | central gray, alpha part |
| CGPn | central gray of the pons |
| Ci | caudal interstitial nucleus of the medial longitudinal fasciculus |
| CIC | central nucleus of the  inferior colliculus |
| Cir | circular nucleus |
| CL | centrolateral thalamic nucleus |
| Cl | claustrum |
| Cli | caudal linear nucleus of the raphe |
| CM | central medial thalamic nucleus |
| CnF | cuneiform nucleus |
| CPu | caudate putamen (striatum) |
| Cu | cuneate nucleus |
| Cx | cerebral cortex |
| CxA | cortex-amygdala transition zone |
| DC | dorsal cochlear nucleus |
| DCIC | dorsal cortex of the  inferior colliculus |
| DEn | dorsal endopiriform nucleus |
| Dk | nucleus of Darkschewitsch |
| DLG | dorsal lateral geniculate nucleus |
| DLL | dorsal nucleus of the  lateral lemniscus |
| DLPAG | dorsolateral periaqueductal gray |
| DM | dorsomedial hypothalamic nucleus |
| DMC | dorsomedial hyptohalamic nucleus, compact part |
| DMPAG | dorsomedial periaqueductal gray |
| DMPn | dorsomedial pontine nucleus |
| DMTg | dorsomedial tegmental area |
| DMSp5 | dorsomedial spinal trigeminal nucleus |
| DP | dorsal peduncular cortex |
| DpG | deep gray layer of the  superior colliculus |
| DPGi | dorsal paragigantocellular nucleus |
| DpMe | deep mesencephalic nucleus |
| DPO | dorsal periolivary region |
| DpWh | deep white layer of the  superior colliculus |
| DR | dorsal raphe nucleus |
| DRC | dorsal raphe nucleus, caudal part |
| DRD | dorsal raphe nucleus, dorsal part |
| DRI | dorsal raphe nucleus,  interfascicular part |
| DRV | dorsal raphe nucleus, ventral part |
| DRVL | dorsal raphe nucleus,  ventrolateral part |
| DT | dorsal terminal nucleus of the accessory optic tract |
| DTg | dorsal tegmental nucleus |
| DTgC | dorsal tegmental nucleus,  central part |
| DTgP | dorsal tegmental nucleus, pericentral part |
| DTT | dorsal tenia tecta |
| ec | external capsule |
| ECIC | external cortex of the  inferior colliculus |
| Ect | ectorhinal cortex |
| ECu | external cuneate nucleus |
| E/OV | ependymal and subendymal layer/olfactory ventricle |
| EMi | epimicrocellular nucleus |
| EPl | external plexiform layer of the olfactory bulb |
| EPlA | external plexiform layer of the accessory olfactory bulb |
| ERS | epirubrospinal nucleus |
| EW | Edinger-Westphal nucleus |
| EVe | nucleus of origin of efferents of the vestibular nerve |
| ex | extreme capsule |
| f | fornix |
| FC | fasciola cinereum |
| FCd | nucleus accumbens, caudate fundus |
| FPu | nucleus accumbens, putaminal fundus |
| FrA | frontal association cortex |
| Fu | bed nucleus of stria terminalis, fusiform part |
| FVe | F cell group of the vestibular complex |
| Ge5 | gelatinous layer of the caudal spinal trigeminal nucleus |
| Gi | gigantocellular reticular nucleus |
| GiA | gigantocellular reticular nucleus, alpha part |
| GiV | gigantocellular reticular nucleus, ventral part |
| Gl | glomerular layer of the  olfactory bulb |
| GlA | glomerular layer of the accessory olfactory bulb |
| GP | globus pallidus |
| GPe | globus pallidus, external segment |
| GPi | globus pallidus, internal segment |
| Gr | gracile nucleus |
| GrA | granule cell layer of the accessory olfactory bulb |
| GrC | granular layer of the cochlear nuclei |
| GrDG | granular layer of the dentate gyrus |
| GrO | granular cell layer of the  olfactory bulb |
| Gus | gustatory thalamic nucleus |
| HDB | nucleus of the horizontal limb of the diagonal band |
| Hip | hippocampus |
| HTh | hypothalamus |
| I5 | intertrigeminal nucleus |
| I | intercalated nuclei of the amygdala |
| IAD | interanterdorsal thalamic nucleus |
| IAM | interanteromedial thalamic nucleus |
| ic | internal capsule |
| IC | inferior colliculus |
| ICj | islands of Calleja |
| ICjM | islands of Calleja, major island |
| IF | interfascicular nucleus |
| Ig | indusium griseum |
| IG | insular gyrus |
| IGL | intergeniculate leaf |
| IL | infralimbic cortex |
| ILL | intermediate nucleus of the lateral lemniscus |
| IM | intercalated amygdaloid nucleus, main part |
| IMA | intramedullary thalamic area |
| IMD | intermediodorsal thalamic nucleus |
| In | intercalated nucleus of the medulla |
| InC | interstitial nucleus of Cajal |
| InCo | intercollicular nucleus |
| InG | intermediate gray layer of the superior colliculus |
| InM | intermedius nucleus of the medulla |
| InWh | intermediate white layer of the superior colliculus |
| IO | inferior olive |
| IOA | inferior olive, subnucleus A of medial nucleus |
| IOB | inferior olive, subnucleus B of medial nucleus |
| IOBe | inferior olive, beta subnucleus |
| IOC | inferior olive, subnucleus C of medial nucleus |
| IOD | inferior olive, dorsal nucleus |
| IODM | inferior olive, dorsomedial cell group |
| IODMC | inferior olive, dorsomedial cell column |
| IOK | inferior olive, cap of Kooy of the medial nucleus |
| IOM | inferior olive, medial nucleus |
| IOPr | inferior olive, principal nucleus |
| IOVL | inferior olive,  ventrolateral protrusion |
| IP | interpeduncular nucleus |
| IPAC | interstitial nucleus of the posterior limb of the anterior commissure |
| IPACL | intersitital nucleus of the posterior limb of the anterior commissure, lateral part |
| IPACM | intersitital nucleus of the posterior limb of the anterior commissure, medial part |
| IPDM | interpeduncular nucleus, dorsomedial subnucleus |
| IPF | interpeduncular fossa |
| IPL | interpeduncular nucleus,  lateral subnucleus |
| IPl | internal plexiform layer of the olfactory bulb |
| IPR | interpeduncular nucleus,  rostral subnucleus |
| IRt | intermediate reticular nucleus |
| IS | inferior salivatory nucleus |
| KF | Kölliker-Fuse nucleus |
| L1 | cortical layer 1 |
| L2 | cortical layer 2 |
| L3 | cortical layer 3 |
| L4 | cortical layer 4 |
| L5a | cortical layer 5a |
| L5b | cortical layer 5b |
| L6 | cortical layer 6 |
| LA | lateroanterior hypothalamic nucleus |
| La | lateral amygdaloid nucleus |
| LAcbSh | lateral accumbens shell |
| LaDL | lateral amygdaloid nucleus, dorsolateral part |
| LaVL | lateral amygdaloid nucleus, ventromedial part |
| LaVM | lateral amygdaloid nucleus, ventromedial part |
| LC | locus coeruleus |
| Ld | lambdoid septal zone |
| LDDM | laterodorsal thalamic nucleus, dorsomedial part |
| LDTg | laterodorsal tegmental nucleus |
| LDTgV | laterodorsal tegmental nucleus, ventral part |
| LDVL | laterodorsal thalamic nucleus, ventrolateral part |
| LGP | lateral globus pallidus |
| LH | lateral hypothalamic area |
| LHb | lateral habenular nucleus |
| LHbM | lateral habenular nucleus,  medial part |
| Li | linear nucleus of the medulla |
| LM | lateral mammillary nucleus |
| lml | lateral medullary lamina |
| LMol | lacunosum moleculare layer of the hippocampus |
| LO | lateral orbital cortex |
| LOT | nucleus of the lateral olfactory tract |
| LPAG | lateral periaqueductal gray |
| LPB | lateral parabrachial nucleus |
| LPBC | lateral parabrachial nucleus,  central part |
| LPBD | lateral parabrachial nucleus,  dorsal part |
| LPBE | lateral parabrachial nucleus, external part |
| LPBI | lateral parabrachial nucleus,  internal part |
| LPBV | lateral parabrachial nucleus,  ventral part |
| LPGi | lateral paragigantocellular nucleus |
| LPLC | lateral posterior thalamic nucleus, laterocaudal part |
| LPLR | lateral posterior thalamic nucleus, laterorostral part |
| LPMC | lateral posterior thalamic nucleus, mediocaudal part |
| LPMR | lateral posterior thalamic nucleus, mediorostral part |
| LPO | lateral preoptic area |
| LRt | lateral reticular nucleus |
| LRtPC | lateral reticular nucleus, parvicellular part |
| LS | lateral septal nucleus |
| LSD | lateral septal nucleus, dorsal part |
| LSI | lateral septal nucleus,  intermediate part |
| LSO | lateral superior olive |
| LSS | lateral stripe of the striatum |
| LSV | lateral septal nucleus, ventral part |
| LV | lateral ventricle |
| LVe | lateral vestibular nucleus |
| LVPO | lateral periolivary nucleus |
| M2 | secondary motor cortex |
| MA3 | medial accessory  oculomotor nucleus |
| MCLH | magnocellular nucleus of the  lateral hypothalamus |
| MCPC | magnocellular nucleus of the posterior commissure |
| MCPO | magnocellular preoptic nucleus |
| MD | mediodorsal thalamic nucleus |
| MDC | mediodorsal thalamic nucleus, central part |
| MdD | medullary reticular nucleus,  dorsal part |
| MDL | mediodorsal thalamic nucleus, lateral part |
| MDM | mediodorsal thalamic nucleus, medial part |
| MdV | medullary reticular nucleus,  ventral part |
| Me5 | mesencephalic trigeminal nucleus |
| me5 | mesencephalic trigeminal tract |
| MeA | medial amygdaloid nucleus,  anterior part |
| MeAD | medial amygdaloid nucleus,  anterior dorsal part |
| MeAV | medial amygdaloid nucleus, anteroventral part |
| MePD | medial amygdaloid nucleus, posterodorsal part |
| MePV | medial amygdaloid nucleus, posteroventral part |
| MG | medial geniculate nucleus |
| MGD | medial geniculate nucleus,  dorsal part |
| MGM | medial geniculate nucleus,  medial part |
| MGP | medial globus pallidus |
| MGV | medial geniculate nucleus, ventral part |
| MHb | medial habenular nucleus |
| Mi | mitral cell layer of the olfactory bulb |
| MiA | mitral cell layer of the accessory olfactory bulb |
| MiTg | microcellular tegmental nucleus |
| ml | medial lemniscus |
| ML | medial mammillary nucleus, lateral part |
| mlf | medial longitudinal fasciculus |
| MM | medial mammillary nucleus,  medial part |
| mml | medial medullary lamina |
| MMn | medial mammillary nucleus,  median part |
| mMnR | median raphe nucleus, medial part |
| MnA | median accessory nucleus of the medulla |
| MnPO | median preoptic nucleus |
| MnR | median raphe nucleus |
| Mo5 | motor trigeminal nucleus |
| Mol | molecular layer of the  dendate gyrus |
| MPA | medial preoptic area |
| MPB | medial parabrachial nucleus |
| MPBe | medial parabrachial nucleus external part |
| MPOC | medial preoptic nucleus, central part |
| MPOL | medial preoptic nucleus, lateral part |
| MPOM | medial preoptic nucleus, medial part |
| MPT | medial pretectal nucleus |
| MS | medial septal nucleus |
| MT | medial terminal nucleus of the accessory optic tract |
| MTu | medial tuberal nucleus |
| MVe | medial vestibular nucleus |
| MVeMC | medial vestibular nucleus, magnocellular part |
| MVePC | medial vestibular nucleus, parvicellular part |
| MVPO | medioventral periolivary nucleus |
| MZMG | marginal zone of the  medial geniculate |
| NBM | nucleus basalis of Meynert |
| NCS | nucleus centralis superior |
| OB | olfactory bulb |
| OlfA | olfactory area |
| Op | optic nerve layer of the  superior colliculus |
| OPC | oval paracentral thalamic nucleus |
| OPT | olivary pretectal nucleus |
| OT | nucleus of the optic tract |
| P5 | peritrigeminal zone |
| P7 | perifacial zone |
| Pa4 | paratrochlear nucleus |
| Pa5 | paratrigeminal nucleus |
| Pa6 | paraabducens nucleus |
| PaDC | paraventricular hypothalamic nucleus, dorsal cap |
| PAG | periaqueductal gray |
| PaLM | paraventricular hypothalamic nucleus, lateral magnocellular part |
| PaMM | paraventricular hypothalamic nucleus, medial magnocellular part |
| PaMP | paraventricular hypothalamic nucleus, medial parvicellular part |
| PaR | pararubral nucleus |
| PaS | parasubiculum |
| PBG | parabigeminal nucleus |
| PBP | parabrachial pigmented nucleus |
| PBW | parabrachial nucleus waist part |
| PC | paracentral thalamic nucleus |
| PC5 | parvicellular motor  trigeminal nucleus |
| PCGS | paracochlear glial substance |
| PCRt | parvicellular reticular nucleus |
| PCRtA | parvicellular reticular nucleus,  alpha part |
| PDTg | posterodorsal tegmental nucleus |
| Pe | periventricular hypothalamic nucleus |
| PeF | perifornical nucleus |
| PF | parafascicular thalamic nucleus |
| PH | posterior hypothalamic area |
| PIL | posterior intralaminar  thalamic nucleus |
| Pir | piriform cortex |
| PirF | cortex pre-piriformis |
| PL | paralemniscal nucleus |
| PLCo | posterolateral cortical  amygdaloid nucleus (C2) |
| PMCo | posteromedial cortical  amygdaloid nucleus (C3) |
| PMD | premammillary nucleus, dorsal part |
| PMn | paramedian reticular nucleus |
| PMnR | paramedian raphe nucleus |
| PMV | premammillary nucleus, ventral part |
| PN | paranigral nucleus |
| Pn | pontine nuclei |
| PnC | pontine reticular nucleus, caudal part |
| PnO | pontine reticular nucleus, oral part |
| PnR | pontine raphe nucleus |
| PnV | pontine reticular nucleus,  ventral part |
| Po | posterior thalamic nuclear group |
| PoDG | polymorph layer of the  dentate gyrus |
| PoMn | posteromedian thalamic nucleus |
| PoT | posterior thalamic nuclear group, triangular part |
| PP | peripeduncular nucleus |
| PPT | posterior pretectal nucleus |
| PPTG | pedunculopontine tegmental nucleus |
| PPy | parapyramidal nucleus |
| PR | prerubral field |
| Pr5 | principal sensory trigeminal nucleus |
| Pr5DM | principal sensory trigeminal nucleus, dorsomedial part |
| Pr5VL | principal sensory trigeminal nucleus, ventrolateral part |
| PrC | precommissural nucleus |
| PrL | prelimbic cortex |
| PrS | presubiculum |
| PS | parastrial nucleus |
| PSol | parasolitary nucleus |
| PSTh | parasubthalamic nucleus |
| PT | paratenial thalamic nucleus |
| Pu | putamen |
| PuV | ventral area of putamen |
| PV | paraventricular thalamic nucleus |
| PVA | paraventricular thalamic nucleus, anterior part |
| PVP | paraventricular thalamic nucleus, posterior part |
| Py | pyramidal cell layer of the hippocampus |
| R | red nucleus |
| Rbd | rhabdoid nucleus |
| Rad | stratum radiatum of the hippocampus |
| RAmb | retroambiguus nucleus |
| RC | raphe cap |
| RCh | retrochiasmatic area |
| Re | reuniens thalamic nucleus |
| REth | retroethmoid nucleus |
| Rh | rhomboid thalamic nucleus |
| Ri | rostral interstitial nucleus of medial longitudinal fasciculus |
| RI | restroisthmic nucleus |
| RLi | rostral linear nucleus of the raphe |
| RMC | red nucleus, magnocellular part |
| RMg | raphe magnus nucleus |
| Ro | nucleus of Roller |
| ROb | raphe obscurus nucleus |
| RPa | raphe pallidus nucleus |
| RPC | red nucleus, parvicellular part |
| RPF | retroparafascicular nucleus |
| RPO | rostral periolivary region |
| RR | retrorubral nucleus |
| RRF | retrorubral field |
| RRF/A8 | retrorubral fields/A8 dopamine cells |
| RSD | retrosplenial dysgranular cortex |
| RSGa | retrosplenial granular cortex, a part |
| RSGb | retrosplenial granular cortex, b part |
| RSGc | retrosplenial granular cortex, c part |
| Rt | reticular thalamic nucleus |
| RT | reticulotegmental nucleus of the pons |
| RTP | reticulotegmental nucleus of the pons, pericentral part |
| RVL | rostroventrolateral reticular nucleus |
| S | septal area |
| Sag | sagulum |
| Sb | subiculum |
| SC | superior colliculus |
| SCA | subcallosal area |
| SCh | suprachiasmatic nucleus |
| SChDM | suprachiasmatic nucleus, dorsomedial part |
| SChVL | suprachiasmatic nucleus, ventrolateral part |
| SCO | subcommissural organ |
| scp | superior cerebellar peduncle |
| SFi | septofimbrial nucleus |
| SFO | subfornical organ |
| SG | suprageniculate thalamic nucleus |
| SGl | superficial glial zone of the  cochlear nuclei |
| SHi | septohippocampal nucleus |
| SHy | septohypothalamic nucleus |
| SI | substantia innominata |
| SID | substantia innominata, dorsal part |
| SLEA | sublenticular extended amygdala |
| SLEAC | sublenticular extended amygdala, central part |
| SLEAM | sublenticular extended amygdala, medial part |
| SLu | stratum lucidum, hippocampus |
| SM | nucleus of the stria medullaris |
| SMV | superior medullary velum |
| SN | substantia nigra |
| SNC | substantia nigra, compact part |
| SNL | substantia nigra, lateral part |
| SNR | substantia nigra, reticular part |
| SO | supraoptic nucleus |
| Sol | nucleus of the solitary tract |
| sol | solitary tract |
| SolC | nucleus of the solitary tract, commissural part |
| SolCe | nucleus of the solitary tract, central part |
| SolDL | nucleus of the solitary tract, dorsolateral part |
| SolDM | nucleus of the solitary tract, dorsomedial part |
| SolG | nucleus of the solitary tract, gelatinous part |
| SolI | nucleus of the solitary tract, interstitial part |
| SolIM | nucleus of the solitary tract, intermediate part |
| SolM | nucleus of the solitary tract,  medial part |
| SolV | nucleus of the solitary tract,  ventral part |
| SolVL | nucleus of the solitary tract, ventrolateral part |
| Sp5 | spinal trigeminal nucleus |
| sp5 | spinal trigeminal tract |
| Sp5C | spinal trigeminal nucleus,  caudal part |
| Sp5I | spinal trigeminal nucleus,  interpolar part |
| Sp5O | spinal trigeminal nucleus, oral part |
| Sp5ODM | spinal trigeminal nucleus,  oral part, dorsomedial division |
| Sp5OVL | spinal trigeminal nucleus,  oral part, ventrolateral division |
| SPa | subparaventricular zone of the hypothalamus |
| SPF | subparafascicular thalamic nucleus |
| SPFPC | subparafascicular thalamic nucleus, parvicellular part |
| Sph | sphenoid nucleus |
| SPO | superior paraolivary nucleus |
| SPTg | subpedencular tegmental nucleus |
| SpVe | spinal vestibular nucleus |
| st | spinothalamic tract |
| StA | strial part of the preoptic area |
| STh | subthalamic nucleus |
| StHy | striohypothalamic nucleus |
| Su3 | supraoculomotor periaqueductal gray |
| Su3C | supraoculomotor cap |
| Su5 | supratrigeminal nucleus |
| Sub | submedius thalamic nucleus |
| SubB | subbrachial nucleus |
| SubCD | subcoeruleus nucleus, dorsal part |
| SubCV | subcoeruleus nucleus, ventral part |
| SubG | subgeniculate nucleus |
| SubI | subincertal nucleus |
| SuG | superficial gray layer of the  superior colliculus |
| SuM | supramammillary nucleus |
| SuML | supramammillary nucleus,  lateral part |
| SuMM | supramammillary nucleus,  medial part |
| sumx | supramammillary decussation |
| SuVe | superior vestibular nucleus |
| TC | tuber cinereum area |
| Th | thalamus |
| Te | terete hypothalamic nucleus |
| TS | triangular septal nucleus |
| Tu | olfactory tubercle |
| Tz | nucleus of the trapezoid body |
| V1 | primary visual cortex |
| V2ML | secondary visual cortex, mediolateral area |
| V2MM | secondary visual cortex, mediomedial area |
| VA | ventral anterior thalamic nucleus |
| VC | ventral cochlear nucleus |
| VCA | ventral cochlear nucleus,  anterior part |
| VCP | ventral cochlear nucleus,  posterior part |
| VDB | nucleus of the vertical limb of the diagonal band |
| VEn | ventral endopiriform nucleus |
| VL | ventrolateral thalamic nucleus |
| VLG | ventral lateral geniculate nucleus |
| VLGMC | ventral lateral geniculate nucleus, magnocellular part |
| VLGPC | ventral lateral geniculate nucleus, parvicellular part |
| VLL | ventral nucleus of the lateral lemniscus |
| VLPAG | ventrolateral periaqueductal gray |
| VLPO | ventrolateral preoptic nucleus |
| VLTg | ventrolateral tegmental area |
| VM | ventromedial thalamic nucleus |
| VMH | ventromedial hypothalamic nucleus |
| VMHC | ventromedial hypothalamic nucleus, central part |
| VMHDL | ventromedial hypothalamic nucleus, dorsolateral part |
| VMHDM | ventromedial hypothalamic nucleus, dorsomedial part |
| VMHVL | ventromedial hypothalamic nucleus, ventrolateral part |
| VMPO | ventromedial preoptic nucleus |
| VO | ventral orbital cortex |
| VOLT | vascular organ of the  lamina terminalis |
| VP | ventral pallidum |
| VPL | ventral posterolateral  thalamic nucleus |
| VPM | ventral posteromedial  thalamic nucleus |
| VRe | ventral reuniens thalamic nucleus |
| VTM | ventral tuberomammillary nucleus |
| VTA | ventral tegmental area |
| VTg | ventral tegmental nucleus |
| VTRZ | visual tegmental relay zone |
| VTT | ventral tenia tecta |
| X | nucleus X |
| Xi | xiphoid thalamic nucleus |
| Y | nucleus Y |
| ZI | zona incerta |
| ZID | zona incerta, dorsal part |
| ZIV | zona incerta, ventral part |
| ZL | zona limitans |

| **Brain  nuclei** | | **WT** | **3413**  **UBB^+1^I** | | **3413**  **UBB^+1^ D** | | **Brain nuclei** | **WT** | **3413**  **UBB^+1^I** | | **3413**  **UBB^+1^ D** |
| --- | --- | --- | --- | --- | --- | --- | --- | --- | --- | --- | --- |
| **Prosencephalon** | | | | | | | BAC | − | + + | + | |
| Telencephalon | | | | | | | Cir | − | + | | + |
| Cl | | − | | + + | | + + | IPAC | − | + | | + + |
| f | | − | | − | | − | IPACL | − | + | | + + |
| PSTh | | − | | + + | | + | IPACM | − | + | | + + |
| SFO | | − | | + + | | + + + | LHb | − | + | | + |
| SI | | − | | + | | + | LHbM | − | + | | + |
| SID | | − | | + | | + + | MCPC | − | − | | − |
| VP | | − | | + | | + + | MHb | − | + | | + |
| *Septal area* | | | | | | | REth | − | + | | + |
| HDB | − | | | + | | + | SM | − | + | | + |
| Ld | − | | | − | | − | VOLT | − | + + | | + |
| LS | − | | | + + | | + + + | *Preoptic nuclei* | | | | |
| LSD | − | | | + + | | + + + | ADP | − | + + | | + |
| LSI | − | | | + + | | + + + | LPO | − | + | | + |
| LSV | − | | | + + | | + + + | MCPO | − | + | + | |
| MS | − | | | − | | − | MnPO | − | + + | | + |
| SFi | − | | | + + | | + | MPA | − | + | | + + |
| TS | − | | | + + | | + + | MPOC | − | + | | + + |
| VDB | − | | | + | | + | MPOL | − | + | | + + |
| *Bed nucleus of stria terminalis* | | | | | | | MPOM | − | + | | + + |
| BST | − | | | + | | + | PS | − | + | | + + |
| BSTIA | − | | | + | | + + | StA | − | + + | | + |
| BSTL | − | | | + + | | + | SO | − | + | | + + |
| BSTLD | − | | | + + | | + | VLPO | − | + | | + |
| BSTLI | − | | | + + | | + + | VMPO | − | + + | | + + |
| BSTLJ | − | | | + + | | + | *Geniculate nuclei* | | | | |
| BSTLP | − | | | + + | | + + | DLG | − | + | + | |
| BSTLV | − | | | + | | + | IGL | − | + | | + |
| BSTMA | − | | | + | | + + | SubG | − | + | + | |
| BSTMPI | − | | | + + | | + + | VLG | − | + | | + + |
| BSTMPL | − | | | + | | + | VLGMC | − | + | | + |
| BSTMPM | − | | | + + | | + + | VLGPC | − | + | | + |
| BSTMV | − | | | + | | + + | *Subthalamic nuclei* | | | | |
| BSTS | − | | | − | | − | A13 | − | + | | + |
| Fu | − | | | + + | | + | SubI | *−* | + | | + |
| Diencephalon | | | | | | | ZI | − | + | + | |
| AC | − | | | − | | − | ZID | − | + | | + |
| AVPe | − | | | + + | | + | ZIV | − | + | | + |

| **Brain**  **nuclei** | **WT** | **3413**  **UBB^+1^I** | **3413**  **UBB^+1^ D** | **Brain nuclei** | **WT** | **3413**  **UBB^+1^I** | | **3413**  **UBB^+1^ D** |
| --- | --- | --- | --- | --- | --- | --- | --- | --- |
| *Thalamus nuclei* | | | | SPF | − | + | | + |
| AD | − | + | + + | SPFPC | − | + + | | + |
| AM | − | + | + + | Sub | − | + | | + |
| AMV | − | + | + | VA | − | + | | + |
| Ang | − | + | + | VL | − | + | | + |
| AV | − | + | + + | VM | − | + | | + |
| AVDM | − | + | + + | VPL | − | + | | + |
| AVVL | − | + + | + + | VPM | − | + | | + |
| CL | − | + | + | VRe | − | + | | + |
| CM | − | + | + | Xi | − | + + | | + |
| Gus | − | + | + | ZL | − | + | | + |
| IAD | − | + | + | *Hypothalamus nuclei* | | | | |
| IAM | − | + | + | A12 | − | + + | | + + |
| IMA | − | + | + | AHA | − | + | | + |
| IMD | − | + | + | AHC | − | + | | + |
| LDDM | − | + | + | AHP | − | + | + + | |
| LDVL | − | + | + | Arc | − | + + | | + + |
| LPLC | − | + + | + | ArcD | − | + + | | + + |
| LPLR | − | + | + | ArcL | − | + + | | + + |
| LPMC | − | + | + | ArcLP | − | + + | | + |
| LPMR | − | + | + | ArcMP | − | + + | | + + |
| MD | − | + | + | DM | − | + + | | + |
| MDC | − | + | + | DMC | − | + + | | + |
| MDL | − | + | + | LA | − | + | | + + |
| MDM | − | + | + | LH | − | + | | + |
| OPC | − | + | + | MCLH | − | + | | + |
| PC | − | + | + | MTu | − | + + | | + |
| PF | − | + | + | PaDC | − | + | + | |
| PIL | − | + | + | PaLM | − | + | | + |
| Po | − | + | + | PaMM | − | + | | + |
| PoMn | − | + | + | PaMP | − | + + | | + + |
| PoT | − | + + | + | Pe | − | + | | + |
| PT | − | + | + | PeF | − | + + | | + |
| PV | − | + | + + | PH | − | + | | + |
| PVA | − | + + | + + | RCh | − | + + | | + + |
| PVP | − | + + | + + | SCh | − | + + | | + |
| Re | − | + | + | SChDM | − | + + | | + + |
| Rh | − | + | + | SChVL | − | + | | + + |
| Rt | − | + | + | SHy | − | + + | | + |
| SG | − | + | + | SPa | − | + + | | + + |
| **Brain**  **nuclei** | **WT** | **3413**  **UBB^+1^I** | **3413**  **UBB^+1^ D** | **Brain nuclei** | **WT** | **3413**  **UBB^+1^I** | | **3413**  **UBB^+1^ D** |
| StHy | − | + + | + + | *Tegmentum* | | | | |
| TC | − | − | − | 3N | − | + | | + + |
| Te | − | + + | + | 4N | − | − | | − |
| VMH | − | + + | + + | 3PC | − | + | | + + |
| VMHC | − | + + | + + | ATg | − | − | − | |
| VMHDL | − | + | + + | Ci | − | + | | + |
| VMHDM | − | + | + | CG | − | − | | − |
| VMHVL | − | + | + | CGA | − | + | | + + |
| *Mammillary bodies* | | | | CnF | − | + | | + |
| LM | − | + + | + | Dk | − | + + | | + |
| ML | − | + | + | DLPAG | − | + | | + |
| MM | − | + + | + + | DMPAG | − | + | | + + |
| MMn | − | − | − | DMTg | − | + | | + |
| PMD | − | − | − | DT | − | + | | + |
| PMV | − | + + | + | DTgC | − | + | | + |
| VTM | − | + + | + | DTgP | − | + | | + |
| SuM | − | + + | + + | Emi | − | + | | + |
| SuML | − | ++ | + | EW | − | + | | + |
| SuMM | − | + | + | IF | − | + | | + |
| sumx | − | + | + | InC | − | + + | | + |
| **Mesencephalon** | | | | IP | − | + | | + + |
| *Tectum* | | | | IPDM | − | + | | + + |
| APT | − | + | + | IPL | − | + | | + |
| APTD | − | + | + | IPR | − | + | | + |
| APTV | − | − | − | LPAG | − | + | | + |
| DpG | − | + | + | MA3 | − | − | | − |
| DpMe | − | + | + | MiTg | − | + | | + |
| DpWh | − | + | + | Me5 | − | + | | + |
| InCo | − | + | + | MT | − | + | | + |
| InG | − | + | + | Pa4 | − | + + | | + + |
| InWh | − | + | + | PAG | − | + | | + |
| MPT | − | − | − | PaR | − | + | | + |
| Op | − | + | + | PBP | − | + | | + + |
| OPT | − | + | + | PDTg | − | + | | + + |
| OT | − | + | + | PN | − | + + | | + |
| PBG | − | + | + | PR | − | + | | + |
| PPT | − | + | + | PrC | − | + | | + |
| SCO | − | + | + | R | − | − | | − |
| SubB | − | + | + + | Ri | − | − | | − |
| SuG | − | + | + | RMC | − | − | | − |
| **Brain**  **nuclei** | **WT** | **3413**  **UBB^+1^I** | **3413**  **UBB^+1^ D** | **Brain nuclei** | **WT** | **3413**  **UBB^+1^I** | | **3413**  **UBB^+1^ D** |
| RPC | − | − | − | P7 | − | + + | | + |
| RR | − | + | + | Pa6 | − | − | | − |
| RRF | − | + + | + | PC5 | − | − | | − |
| Sag | − | − | − | PL | − | + | | + |
| SNC | − | + | + + | Pn | − | + | | + |
| SNL | − | + | + | PnC | − | + + | | + |
| SNR | − | − | − | PnO | − | + | | + |
| SPtg | − | + | + | PnV | − | + | | + |
| Su3 | − | + | + | PPTG | − | + | | + |
| Su3C | − | + | + | Pr5 | − | − | | − |
| SubB | − | + | + + | Pr5DM | − | + + | | + |
| VLPAG | − | + | + + | Pr5VL | − | − | | − |
| VTA | − | + | + | RPO | − | + | | + |
| VTg | − | − | − | RtTg | − | − | | − |
| VTRZ | − | − | − | RtTgP | − | + | | + |
| **Rhombencephalon** | | | | SMV | − | − | | − |
| Metencephalon | | | | Sph | − | + | | + + |
| *Pons* | | | | SPO | − | + + | | + |
| 6N | − | − | − | Su5 | − | + | | + |
| 7N | − | + | + | SubCD | − | − | | − |
| A5 | − | + + | + | SubCV | − | + | | + |
| A7 | − | − | − | VLL | − | + | | + |
| Asc5 | − | − | − | VLTg | − | + | | + |
| Asc6/7 | − | + | + | Myelencephalon | | | | |
| B9 | − | − | − | *Medulla* | | | | |
| Bar | − | + | + + | 12N | − | − | | − |
| CGPn | − | + | + | A1 | − | − | | − |
| DLL | − | + | + | A2 | − | − | − | |
| DMPn | − | − | − | Amb | − | − | − | |
| DPO | − | + | + | B4 | − | − | | − |
| ERS | − | + | + | Bo | − | − | | − |
| I5 | − | + | + | C1 | − | + | | + |
| ILL | − | + | + | C2 | − | + + | | + + |
| KF | − | − | − | C3 | − | − | | − |
| LDTg | − | + | + | Cu | − | + | | + |
| LDTgV | − | + | + | CeCv | − | − | | − |
| LVPO | − | − | − | DMSp5 | − | + | | + |
| Mo5 | − | + | + | DPGi | − | + | | + |
| MVPO | − | + | + | ECu | − | + | | + |
| P5 | − | + | + | EVe | − | − | | − |
| **Brain**  **nuclei** | **WT** | **3413**  **UBB^+1^I** | **3413**  **UBB^+1^ D** | **Brain nuclei** | **WT** | **3413**  **UBB^+1^I** | | **3413**  **UBB^+1^ D** |
| FVe | − | − | − | RAmb | − | − | | − |
| Ge5 | − | + | + + | Rbd | − | + | | + + |
| Gi | − | + | + | Ro | − | + | | + |
| GiA | − | + | + | RVL | − | − | | − |
| GiV | − | − | − | SGl | − | − | | − |
| Gr | − | + | + | Sp5 | − | + + | | + |
| In | − | − | − | Sp5C | − | + | | + |
| InM | − | − | − | Sp5I | − | + | | + |
| IO | − | − | − | Sp5O | − | − | | − |
| IOA | − | − | − | SP5ODM | − | + | | + |
| IOB | − | − | − | SP5OVL | − | + | | + |
| IOBe | − | − | − | SpVe | − | + | | + |
| IOC | − | − | − | SuVe | − | − | | − |
| IOD | − | − | − |  |  |  | |  |
| IODM | − | − | − |  |  |  | |  |
| IODMC | − | − | − |  |  |  | |  |
| IOK | − | − | − |  |  |  | |  |
| IOM | − | + | + |  |  |  | |  |
| IOPr | − | + | + |  |  |  | |  |
| IOVL | − | − | − |  |  |  | |  |
| IRt | − | + | + |  |  |  | |  |
| IS | − | − | − |  |  |  | |  |
| Li | − | − | − |  |  |  | |  |
| LPGi | − | + | + |  |  |  | |  |
| LRt | − | − | − |  |  |  | |  |
| LRtPC | − | − | − |  |  |  | |  |
| LVe | − | − | − |  |  |  | |  |
| MdD | − | + | + |  |  |  | |  |
| MdV | − | + | + |  |  |  | |  |
| MnA | − | − | − |  |  |  | |  |
| MVe | − | + | + |  |  |  | |  |
| MVeMC | − | + | + |  |  |  | |  |
| MVePC | − | + | + |  |  |  | |  |
| Pa5 | − | + + | + |  |  |  | |  |
| PCGS | − | − | − |  |  |  | |  |
| PCRt | − | + | + |  |  |  | |  |
| PCRtA | − | + | + |  |  |  | |  |
| PMn | − | − | − |  |  |  | |  |
| PPy | − | − | − |  |  |  | |  |

*- no immunoreactivity; + low I or D; moderate I or D; +++ high I or D; ++++ very high I or D.*

**Supplementary figures**

**
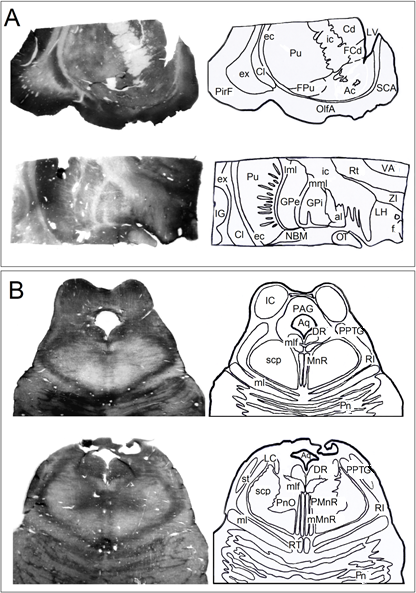
**

**Figure S1.** Representative low-magnification photomicrographs of 50 μm-thick coronal sections from the basal ganglia (**A**) and the mesencephalon (**B**) with the corresponding schematic overview of the neuroanatomical substrates. Uppercase is used to indicate nuclei, whereas lowercase refers to white matter tracts. Abbreviations: ac, nucleus accumbens; al, ansa lenticularis; Aq, cerebral aqueduct; Cd, caudate nucleus; Cl, claustrum; DR, dorsal raphe nucleus; ec, external capsule; ex, extreme capsule; FCd, nucleus accumbens, caudate fundus; FPu, nucleus accumbens, putaminal fundus; f, fornix; GPe, globus pallidus, external segment; GPi, globus pallidus, internal segment; ic, internal capsule; IC, inferior colliculus; IG, insular gyrus; LC, locus coeruleus; LH, lateral hypothalamic area; lml, lateral medullary lamina; LV, lateral ventricle; ml, medial lemniscus; mlf, medial longitudinal fasciculus; mml, medial medullary lamina; MnR, median raphe nucleus; mMnR, median raphe nucleus, medial part; NBM, nucleus basalis of Meynert; OlfA, olfactory area; OT, optic tract; PAG, periaqueductal gray; PirF, cortex pre-piriformis; PMnR, median raphe nucleus, paramedian part; Pn, pontine nuclei; PnO, pontine reticular nucleus, oral part; PPTG, peduncolotegmental nucleus; Pu, putamen; RI, restroisthmic nucleus; RT, reticulotegmental nucleus; Rt, reticular thalamic nucleus; SCA, subcallosal area; scp, superior cerebellar peduncle; st, spinothalamic tract; VA, ventral anterior thalamic nucleus; ZI, zona incerta.
